# Pregnant women admitted to hospital with covid-19 in 10 European countries: individual patient data meta-analysis of population based cohorts in International Obstetric Survey Systems

**DOI:** 10.1136/bmjmed-2023-000733

**Published:** 2024-12-12

**Authors:** Hilde Marie Engjom, Odette de Bruin, Rema Ramakrishnan, Anna J M Aabakke, Outi Äyräs, Catherine Deneux-Tharaux, Serena Donati, Marian Knight, Eva Jonasdottir, Teresia Svanvik, An Vercoutere, Nicola Vousden, Kitty WM Bloemenkamp, on behalf of the INOSS covid-19 in pregnancy working group, An Vercoutere

**Affiliations:** 1 Division of Mental and Physical Health, Norwegian Institute of Public Health, Bergen, Norway; 2 National Perinatal Epidemiology Unit, University of Oxford, Oxford, UK; 3 Department of Obstetrics, Birth Centre Wilhelminas Children's Hospital, Division Woman and Baby, University Medical Centre Utrecht, Utrecht, Netherlands; 4 Department of Biostatistics and Research support, Julius Centre for Health Sciences and Primary Care, University Medical Centre, Utrecht University, Utrecht, Netherlands; 5 Department of Obstetrics and Gynaecology, Copenhagen University Hospital-Holbæk, Kobenhavn, Denmark; 6 Department of Clinical Medicine, University of Copenhagen, Kobenhavn, Denmark; 7 Department of Obstetrics and Gynaecology, Helsinki University Central Hospital, Helsinki, Finland; 8 Faculty of Medicine, University of Helsinki, Helsinki, Finland; 9 Obstetric, Perinatal and Paediatric Epidemiology, Centre for Research on Epidemiology and Statistics, National Institute of Health and Medical Research, Paris, France; 10 Institute Santé des Femmes, Universite Paris Cite, Paris, France; 11 National Centre for Disease Prevention and Health Promotion, Istituto Superiore di Sanità - Italian National Institute of Health, Rome, Italy; 12 Department of Obstetrics and Gynaecology, Landspitali National University Hospital of Iceland, Reykjavik, Iceland; 13 Department of Obstetrics and Gynaecology, Sahlgrenska University Hospital, Goteborg, Sweden; 14 Department of Obstetrics and Gynaecology, Hopital Erasme, Bruxelles, Belgium; 15 Chair, International Obstetric Survey Systems, Utrecht, Netherlands

**Keywords:** COVID-19, Pregnancy complications, Public health

## Abstract

**Objectives:**

To assess the incidence of hospital admissions for covid-19 disease in pregnant women, severity of covid-19 disease, and medical treatment provided to pregnant women with moderate to severe covid-19 during the first 10 months of the pandemic.

**Design:**

Individual patient data meta-analysis of population based cohorts in International Obstetric Survey Systems.

**Setting:**

10 European countries with national or regional surveillance within the International Obstetric Survey Systems (INOSS) collaboration using aligned definitions and case report forms: Belgium, France (regional), Italy, the Netherlands, Denmark, Finland, Iceland, Norway, Sweden (regional), and the UK. The dominant variant of the SARS-CoV-2 virus was the wild-type variant in all countries during the study period (1 March 2020 to 31 December 2020).

**Participants:**

The source population was 1.7 million women giving birth (maternities) from 1 March 2020 to 31 December 2020; pregnant women were included if they were admitted to hospital and had a positive polymerase chain reaction test for the SARS-CoV-2 virus ≤7 days before hospital admission, during admission, or up to two days after giving birth. We further categorised the hospital admission in two groups; covid-19 admission (hospital admission due to covid-19 or with reported symptoms of covid-19 disease) or non-covid-19 admission (admission to hospital for obstetric healthcare or no symptoms of covid-19 disease).

**Main outcome measures:**

Incidence of hospital admissions for covid-19 per 1000 maternities, frequency of moderate to severe covid-19 disease, and number of women who received specific medical treatment for SARS-CoV-2 infection. Moderate to severe covid-19 disease was defined as maternal death, admission to an intensive care unit, or need for respiratory support.

**Results:**

Among 1.7 million maternities, 9003 women were included in the study: 2350 (26.1%) were admitted to hospital because of covid-19 disease or had symptoms of disease. The pooled incidence of hospital admissions for covid-19 per 1000 maternities was 0.8 (95% confidence interval (CI) 0.5 to 1.2, τ^2^=0.44), ranging from no hospital admissions in Iceland to 1.7 in France and 1.9 in the UK. 13 women died due to covid-19. Among 2219 women admitted to hospital for covid-19 in countries with complete information on respiratory support, 820 women (39.5%, 95% CI 34.6% to 44.4%, τ^2^=0.02) had moderate to severe covid-19 disease. At most, 16.8% (95% CI 7.7% to 32.9%, I^2^=81.8%, τ^2^=0.7) of women with moderate to severe covid-19 received specific medical treatment for SARS-CoV-2 infection with corticosteroids, although 66.6% (59.4% to 73.2%, I^2^=50.1, τ^2^=0.06) were given thromboprophylaxis.

**Conclusions:**

Population based surveillance in 10 European countries during the first 10 months of the covid-19 pandemic showed variations in the risk of hospital admissions for covid-19 in pregnant women. This finding indicates that national public health policies likely had a substantial and previously unrecognised role in protecting pregnant women. Few pregnant women with moderate to severe covid-19 were given specific medical treatment for SARS-CoV-2 disease, even when there were no or minor safety concerns. Lessons for future pandemics include the importance of rapid, robust surveillance systems for maternal and perinatal health, and of including use for pregnant women early in the development and testing of medicines and vaccines for public health emergencies.

**WHAT IS ALREADY KNOWN ON THIS TOPIC**

**WHAT THIS STUDY ADDS**

- Large variations in the incidence of hospital admissions for covid-19 were seen in different European countries with comparable public obstetric healthcare systems, suggesting that public health policies, such as early suppression of viral transmission, might protect pregnant women from severe disease
- The study highlighted persistent medical under-treatment of pregnant women with moderate to severe disease during the initial pandemic waves, including when there were few safety concerns about the use of available medicines

**HOW THIS STUDY MIGHT AFFECT RESEARCH, PRACTICE, OR POLICY**

- Investment in rapid, robust surveillance is urgently needed to monitor the effects on pregnant women and their infants during outbreaks or other health emergencies
- Earlier availability of population based data of high quality could have informed health policies, provided better information for pregnant women and their partners, and informed advice and medical care provided by clinicians
- Further joint efforts are needed to address the substantial inequity in receipt of evidence based treatment by pregnant women in public health emergencies

## Introduction

 The covid-19 pandemic evolved over multiple phases and was a worldwide health challenge for more than three years. Currently, the emphasis globally is evaluation and preparedness for future emergencies.^1^,^4^ In hindsight, covid-19 went on to become the most common cause of maternal deaths in the UK during the period 2019-21.^5^ Public health interventions to limit viral transmission are continously discussed and evaluated, along with personal protection such as vaccination and early treatment for people at risk of severe disease.^6^ Europe had two waves of the pandemic during 2020,^7^ and strategies to contain transmission differed across European countries, such as early suppression (in Denmark, Finland, Iceland, Norway, and Italy),^8^,^10^ mitigation (in Sweden and the UK),^11 12^ late suppression (in France),^13^ and intermediate solutions (in the Netherlands and Belgium).^7 14^

According to surveillance by the World Health Organization, the pandemic epicentre moved from China to Europe to the US during 2020, but few of the first studies of covid-19 in pregnancy were from Europe, and even fewer studies were population based. The WHO living systematic review of clinical manifestations, risk factors, and maternal and perinatal outcomes of covid-19 disease in pregnancy summarised the literature published up to 27 April 2021.^15^ Most of the studies included in the review were from countries outside of Europe, were hospital based, and often lacked clear discrimination between hospital admissions for covid-19 and admissions for other pregnancy related healthcare, such as obstetric emergencies or labour. Although many studies looked at individual maternal risk factors, limited information was provided about the source populations or population denominator, and therefore comparison of the incidence of covid-19 in pregnancy in different countries has been lacking.

Evidence from earlier outbreaks, epidemics, and pandemics indicated that pregnant women were susceptible to severe outcomes, and frequently could not access medical treatment prescribed to women who were not pregnant or access vaccines.^16^ Experiences from the UK Obstetric Surveillance System (UKOSS) during the 2009-10 H1N1 pandemic showed that early antiviral treatment in pregnant women admitted to hospital was associated with a reduced odds of admission to an intensive care unit, but few pregnant women started treatment before hospital admission.^17^ At the start of the covid-19 pandemic, knowledge about effective treatment of the SARS-CoV-2 virus was lacking, particularly for pregnant women, and in national studies the proportion of pregnant women who received medical treatment was low.^18^,^20^ Most of the larger international treatment trials, such as WHO Solidarity Therapeutics Trial, initially excluded pregnant women, with the RECOVERY (Randomised Evaluation of Covid-19 Therapy) trial in the UK as one of the exceptions.^21^ Consequently, little information was available about the use of medical treatments directed at the SARS-CoV-2 disease in pregnant women.

The aim of our study was to describe the incidence of hospital admissions for covid-19 disease in pregnant women, severity of covid-19 disease, and medical treatment provided to pregnant women with moderate to severe covid-19 disease, in 10 European countries with different public health containment strategies. Our ultimate aim was to inform future responses to similar situations.

## Methods

### Study population

The source population was women who gave birth (maternities) from 1 March to 31 December 2020, based on national population sources or hospital records. Information about self-reported gender was not collected. The population based surveillance was implemented nationally in Belgium, Denmark, Finland, Iceland, Italy, the Netherlands, Norway, and the UK, and regionally in France, where 60% of the maternity population was included in this study, and in Sweden, where three regional university hospitals and two regions represented 31% of the national maternities (online supplemental table 1). Online supplemental material and a previous publication have further details about the study populations and organisation of surveillance, including testing strategies.^22^ All participating countries had public healthcare for pregnant women, with tax based or insurance based funding.

Pregnant women were included in the study if they were admitted to hospital between 1 March and 31 December 2020 with a positive reverse transcription polymerase chain reaction (PCR) test for the the SARS-CoV-2 virus ≤7 days before or during admission in pregnancy, and up to two days after giving birth. Further categorisation was based on the cause of admission, or covid-19 symptoms if the cause of admission was not known. The covid-19 admission group comprised pregnant women admitted to hospital for covid-19 or those who were reported to have symptoms of covid-19 disease. The non-covid-19 admission group was women admitted to hospital for healthcare not related to covid-19, such as obstetric emergencies or labour, or who were reported to be asymptomatic.

### Outcomes

The primary outcomes were incidence of hospital admissions for covid-19, frequency of moderate to severe covid-19, and frequency of medical treatments specific to covid-19 in pregnant women. Moderate to severe covid-19 was defined as maternal death, admission to an intensive care unit for covid-19, or respiratory support with either oxygen supplementation, high flow nasal oxygen or continuous positive airway pressure, mechanical ventilation, or extracorporeal membrane oxygenation. Only extracorporeal membrane oxygenation, mechanical ventilation, or continuous positive airway pressure was recorded in Belgium, and not oxygen supplementation, and in Sweden only mechanical ventilation or extracorporeal membrane oxygenation was recorded.

Medical treatment specific to covid-19 was defined as one or more of the following: antiviral treatment (oseltamivir, lopinavir-ritonavir, or remdesivir), hydroxychloroquine, corticosteroids for a maternal indication (any treatment with steroids with a stated maternal indication, or treatment with more than the two doses usually recommended for a fetal indication in preterm birth^23^), low molecular weight heparin for thromboprophylaxis or for treatment of thromboembolism, and other covid-19 specific medical treatments, such as anti-interleukin 6 (eg, tocilizumab) and intravenous immunoglobulin G. Use of antibiotics for any indication was also reported but included all indications and was not limited to azithromycin directed at covid-19 disease.

Secondary outcomes for women were preterm birth before 37 weeks' gestational age and mode of birth (vaginal birth, assisted vaginal birth, or caesarean birth). Caesarean births also included indications unrelated to covid-19 disease. Discriminating between spontaneous preterm birth and medically indicated preterm birth was not possible because the clinical definitions varied between countries (eg, preterm rupture of membranes and subsequent medical indication for induction). Secondary perinatal outcomes were stillbirth, neonatal death before discharge after birth, and admission to a neonatal unit.

Descriptive characteristics included as covariates were gestational age at admission, parity (number of previous births at gestational ages ≥22 weeks or ≥24 weeks in the UK), maternal age at admission, obesity (body mass index ≥30), non-European migrant or ethnic minority background, chronic disease before pregnancy (diabetes or hypertension), and diseases in pregnancy (gestational diabetes and pre-eclampsia). Ethnic minority or non-European migrant background was combined because the availability of information varied across countries. Some countries do not register ethnic group, whereas other countries had no information about the mother's country of birth. Non-European migrant background was defined as the mother's country of birth outside of Europe in all countries except the UK. In the UK, ethnic minority background was defined by self-reported ethnicity as black, Asian, mixed, or other ethnic backgrounds. The respective comparison groups were European born mothers or women with an ethnic group categorised as white.

### Statistical analyses

Because reliable estimation of the number of pregnant women in a population is difficult, we used pregnant women giving birth during the study period as the denominator population. Denominator sources were national registries, population statistics, or hospital records, for total maternities and average number of maternities per month.

We conducted a one-stage individual participant data meta-analysis of binomial data to estimate the pooled incidence of hospital admissions for covid-19 per 1000 maternities and the pooled proportion of moderate to severe infection and medical treatment for pregnant women.^24^ For this purpose, we used the metapreg Stata package for meta-analysis of binomial data with hierarchical modelling,^25^ with Wilson's estimation of confidence intervals (CI) due to small proportions,^26^ and specifying the power option in Stata to obtain estimates on a per 1000 or per cent scale.

Heterogeneity was assessed with the I^2^ statistic, τ^2^, and prediction intervals. I^2^ measures the percentage of total variance in the study estimates that is caused by heterogeneity between studies. The I^2^ statistic used in metapreg is based on a population parameter I^2^ statistic proposed by Zhou and Dendukuri for binomial normal data.^27^ This statistic takes into account the mean-variance relation across studies and the sample size of studies included in the meta-analysis. Therefore, it tends to perform better than the I^2^ statistic proposed by Higgins and Thompson.^28^ Prediction interval as a measure of heterogeneity denotes the range of probability that can be expected in a new study that is similar to the studies included in the meta-analysis.^29^ We grouped incidence by admission group (covid-19 or non-covid-19). Because of the heterogeneity in the group of non-covid-19 admissions and to avoid misclassification of severe outcomes related to obstetric emergencies in asymptomatic women, further analyses of covid-19 severity, medical treatment, and secondary maternal and perinatal outcomes were reported for women in the covid-19 admission group only.

Sensitivity analyses of incidence were performed by including only countries with national data (excluding regional data from Sweden and France), and also by using proportional meta-analysis by Freeman-Tukey double arcsine transformation (metaprop) and Wilson's estimation of confidence intervals so that the results could be compared with "metapreg."^25 30^ Among pregnant women in the covid-19 admission group in countries where complete information about respiratory support was available (excluding Belgium and Sweden), the risk ratio for moderate to severe covid-19 was estimated grouped by selected risk factors identified from the literature and in the national data^1518 31^,^34^: maternal age (≥35 years *v* <35 years), obesity (obese *v* not obese), and migrant or ethnic minority background (maternal country of birth outside Europe *v* European born, or ethnic minority (black, Asian, mixed, or other ethnic) background *v* white) using a two stage meta-analysis with the random effect maximum likelihood model and the Hartung-Knapp-Sidik-Jonkman variance estimator.^35 36^ The two stage model was used for this meta-analysis because of national data protection regulations that restricted international sharing of individual health data. Also, because of the small numbers of events and concerns about identifiability, aggregate data from Denmark, Finland, and Norway were combined into one geographic category.

Not all countries had sufficient events to compute adjusted risk estimates. Therefore, sensitivity analyses of adjusted risk ratios were performed with the largest datasets (France, Italy, and the UK). The risk ratio associated with age ≥35 years was adjusted for non-European migrant or ethnic minority background, obesity, and hypertension and diabetes before pregnancy. The risk ratio associated with obesity was adjusted for age (continuous), and hypertension and diabetes before pregnancy. The risk ratio for moderate to severe covid-19 in women from a non-European migrant or ethnic minority background was adjusted for age (continuous) only. The pooled proportions of medical treatments (%) in women with moderate to severe infection were estimated with a meta-analysis of binomial data, as described above, with no sensitivity analyses. Meta-analysis of the observational national studies was registered in EU PAS (EUPAS40489).

### Patient and public involvement

No patients or public representatives were involved in the meta-analysis because the international collaboration group did not have any patient or public representatives. Several of the participants in INOSS have national user representatives providing input to study planning or presentation of the national studies. INOSS members will follow their respective plans for dissemination to patients, clinicians, and the public, through public media and online as applicable.

## Results

**Figure 1:**
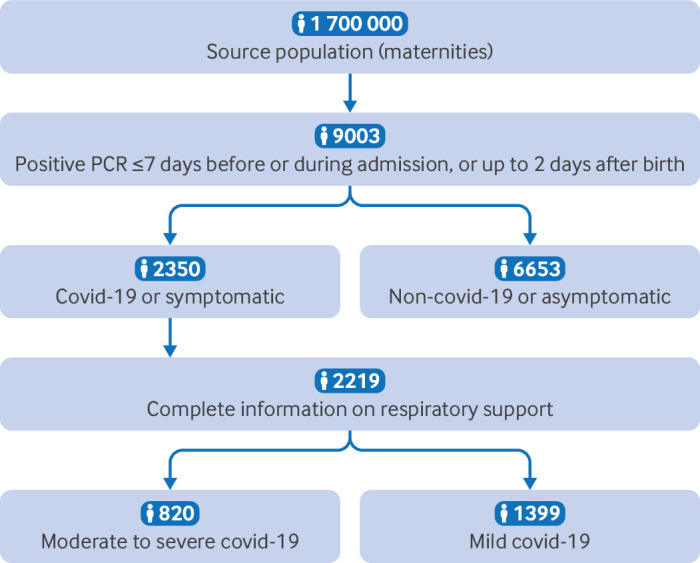
Flowchart for the source population, inclusion criteria, hospital admission group (covid-19 disease or symptoms of covid-19 disease), and severity of covid-19, from 1 March to 31 December 2020, in 10 European countries. PCR=polymerase chain reaction test result. Maternities refers to the number of women giving birth.

Among a source population of 1.7 million women who gave birth between 1 March 2020 and 31 December 2020, 9003 pregnant women were admitted to hospital and had a SARS-CoV-2 infection (online supplemental table 2), of whom 2350 were in the covid-19 admission group (figure 1). In the covid-19 admission group, mean maternal age ranged from 31.0 (standard deviation 4.6) years in Finland to 33.1 (4.6) years in Norway. Median gestational age at admission ranged from 27 (interquartile range 24-36 weeks) in Sweden and 27 (20-32 weeks) in Denmark to 34 (28-38 weeks) in the UK. The proportion of nulliparous women ranged from 20% in Norway and Sweden, to 36.5% in the UK and 37.5% in Finland.

Table 1 shows the characteristics of pregnant women in the covid-19 admission group, by severity of disease (moderate to severe covid-19 or mild covid-19) and country. Women with chronic diseases before pregnancy (hypertension and diabetes) or gestational disease (gestational diabetes and pre-eclampsia) represented <10% of those with moderate to severe covid-19 in all countries, except for gestational diabetes. Online supplemental table 3 provides further details.

**Table 1:** Severity category of covid-19 disease and characteristics in pregnant women admitted to hospital for covid-19 or with symptoms of covid-19 disease, by country, from 1 March to 31 December 2020

Country or region and severity of infection*	No of pregnant women†	Maternal age		Obesity (body mass index ≥30)		Migrant or ethnic minority background‡	
		≥35 years	<35 years	Yes	No	non-European migrant or ethnic minority	Not migrant or white
Belgium:§							
Admissions for covid-19	68	15 (22.1)	53 (77.9)	15 (22.1)	47 (69.1)	NA	NA
France:¶							
Moderate to severe	230	78 (33.9)	152 (66.1)	84 (38.5)	134 (61.5)	92 (42.6)	124 (57.4)
Mild	361	84 (23.3)	277 (76.7)	85 (24.6)	266 (75.8)	139 (41.2)	198 (58.8)
Italy:							
Moderate to severe	122	44 (36.1)	78 (63.9)	33 (28.4)	83 (71.6)	56 (45.9)	66 (54.1)
Mild	172	58 (33.9)	113 (66.1)	15 (9.6)	142 (90.4)	57 (33.1)	115 (66.9)
Netherlands:¶							
Moderate to severe	59	21 (35.6)	38 (64.4)	25 (45.5)	30 (54.5)	30 (60.0)	20 (40.0)
Mild	66	17 (25.8)	49 (74.2)	17 (26.6)	48 (73.8)	31 (51.7)	29 (48.3)
Denmark, Finland, Norway:							
Moderate to severe	25	11 (44.0)	14 (56.0)	13 (52.0)	12 (48.0)	17 (73.9)	6 (26.1)
Mild	37	7 (18.9)	30 (81.1)	11 (30.5)	25 (69.4)	13 (37.1)	22 (62.9)
Sweden:§							
Admissions for covid-19	51	25 (49.0)	26 (51.0)	20 (39.2)	27 (52.9)	33 (64.7)	18 (35.3)
UK:							
Moderate to severe	384	149 (38.8)	235 (61.2)	170 (46.6)	195 (53.4)	219 (58.2)	157 (41.8)
Mild	763	214 (28.1)	549 (71.9)	220 (30.0)	512 (70.0)	385 (51.1)	368 (48.9)

Values are number or number (%) by row (country).

No hospital admissions for covid-19 were recorded in Iceland.

* Moderate to severe covid-19 was defined as maternal death, admission to an intensive care unit, or maximum respiratory support with oxygen, high flow nasal oxygen or continuous positive airway pressure, mechanical ventilation, or extracorporeal membrane oxygenation. Women with mild disease were admitted to hospital for covid-19 or for symptoms of covid-19 disease, but received only supportive treatments, such as intravenous fluids and inhalations, and not respiratory support.

† The sum of categories of characteristics was less than the total in some instances because of missing information. Percentages were calculated based on individuals with known information.

‡ Non-European migrant background was defined as the mother's country of birth outside of Europe in all countries except the UK. In the UK, minority ethnic background was defined as Black, Asian, mixed, or other ethnic backgrounds.

§ Belgium and Sweden did not have detailed data on respiratory support with oxygen and therefore could not discriminate between the severity of covid-19 categories in women admitted to hosptial for covid-19 in the current dataset.

¶ Information about the need for the respiratory support was missing for 10 women in France and two women in the Netherlands.

NA, not available.

**Figure 2:**
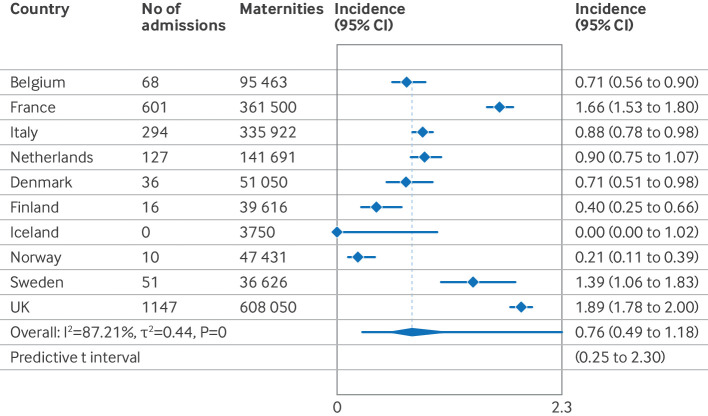
Incidence of hospital admissions for covid-19 disease per 1000 maternities by country, from 1 March to 31 December 2020. Maternities refers to the number of women giving birth. CI=confidence interval

The estimated pooled incidence of hospital admissions for covid-19 disease was 0.8 (95% CI 0.5 to 1.2) per 1000 maternities (I^2^=87.2%, τ^2^=0.44, prediction interval 0.25 to 2.30). Iceland recorded no hospital admissions for covid-19. The incidences in other countries were 0.2 (95% CI 0.1 to 0.4) in Norway, 0.4 (0.3 to 0.7) in Finland, 0.7 (0.6 to 0.9) in Belgium, 0.7 (0.5 to 1.0) in Denmark, 0.9 (1.5 to 1.8) in Italy, 0.9 (0.8 to 1.1) in the Netherlands, 1.7 (1.5 to 1.8) in France, and 1.9 (1.8 to 2.0) in the UK per 1000 maternities (figure 2). When only data from national surveillance were included, the pooled incidence estimate was 0.6 (95% CI 0.4 to 1.0, I^2^=84.5, τ^2^=0.41, prediction interval 0.2 to 2.0). The pooled incidence estimate did not change when we used a model with Freeman-Tukey double arcsine transformation for the meta-analysis of proportions (0.8 per 1000 maternities, 95% CI 0.5 to 1.2, I^2^=98.0%, online supplemental table 4).

**Figure 3:**
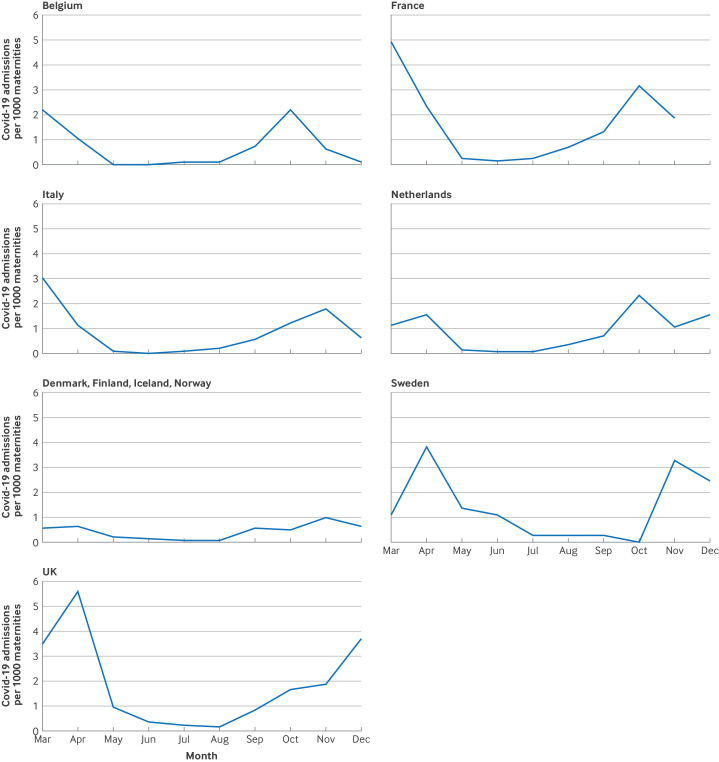
Pregnant women admitted to hospital for covid-19 disease, or symptoms of covid-19 disease if cause of admission was not known, per 1000 maternities, by month of a positive test result for the SARS-CoV-2 virus based on the polymerase chain reaction (PCR) test and by country, from 1 March to 31 December 2020. Data were not available for December 2020 in France. Because of low numbers in individual countries, results from Denmark, Finland, Iceland, and Norway were combined. Maternities refers to the number of women giving birth

The incidence of hospital admissions for covid-19 disease varied over time and by country. Figure 3 shows hospital admissions for covid-19 by the average number of maternities per month, by month of a positive PCR test result and by country. The highest peaks were found during both waves of the pandemic in the French and Swedish regions and in the UK, and the lowest levels in the other four Nordic countries (Denmark, Finland, Iceland, and Norway).

### Moderate to severe covid-19 disease

**Figure 4:**
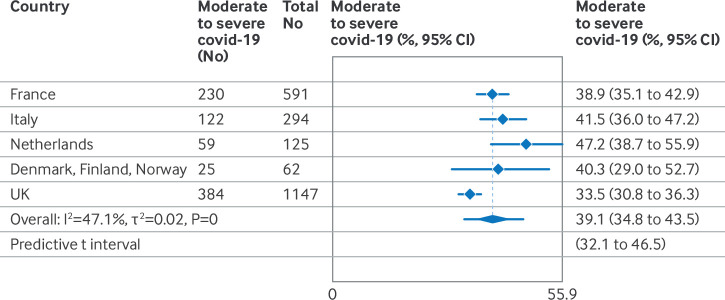
Incidence of moderate to severe covid-19 disease in pregnant women in the covid-19 admission group, by country, from 1 March to 31 December 2020. Moderate to severe covid-19 was defined as maternal death, admission to the intensive care unit, or maximum respiratory support with oxygen by nasal cannula or mask, high flow nasal oxygen or continuous positive airway pressure, mechanical ventilation, or extracorporeal membrane oxygenation. CI=confidence interval

Among 2350 women in the covid-19 admission group, 13 women died and 294 were admitted to an intensive care unit (table 2). Admission to intensive care ranged from 7.8% in Italy to 18.0% and 19.6% in Sweden and France, respectively. Mechanical ventilation or extracorporeal membrane oxygenation was reported for 2-8% of hospital admissions for covid-19, whereas the proportion of women who received respiratory support with oxygen supplementation, continuous positive airway pressure, or high flow nasal cannula ranged from 13% in the UK to 41% in France. Among women in the covid-19 admission group, 39.1% had moderate to severe infection (95% CI 34.8% to 43.5%, I^2^=47.1%, τ ^2^=0.02, prediction interval 32.1 to 46.5), ranging from 33.5% in the UK to 47.2% in the Netherlands (figure 4).

**Table 2:** Maternal deaths, admissions to intensive care unit, and maximum level of respiratory support in pregnant women admitted to hospital for covid-19 disease, by country, from 1 March to 31 December 2020

Country or region	No of hospital admissions for covid-19	No (%) of maternal deaths related to covid-19	No (%) of admissions to ICU	Maximum level of respiratory support (No (%))		
				Mechanical ventilation or ECMO	CPAP or high flow nasal oxygen	Non-rebreather mask or prongs
Belgium	68	0	10 (14.7)	4 (5.9)	3 (4.4)	NA*
France	601	2 (0.3)	108 (18.0)	37 (6.2)	38 (6.3)	210 (35.0)
Italy	294	1 (0.3)	23 (7.8)	12 (4.1)	29 (9.9)	81 (27.6)
Netherlands†	127	0	11 (8.7)	9 (7.1)	18 (14.2))	30 (23.6)
Denmark, Finland, Norway	62	0	7 (11.3)	5 (8.1)	17 (27.4)‡	
Sweden	51	0	10 (19.6)	1 (2.0)	NA§	
UK	1147	10 (0.9)	125 (10.9)	66 (5.7)	43 (3.7)	103 (8.9)

Values are number or number (%) by row (country).

* No information about oxygen supplementation.

† In the Netherlands, information about respiratory support was not available for two women, but all had information about admission to the intensive care unit.

‡ Discriminating between continuous positive airway pressure, high flow nasal oxygen treatment, and oxygen supplementation with a mask or prongs was not possible in Denmark, and consequently these two categories were merged for Denmark, Finland, and Norway

§ No information about respiratory support other than mechanical ventilation or extracorporeal membrane oxygenation was available in Sweden.

CPAP, continuous positive airway pressure; ECMO, extracorporeal membrane oxygenation; ICU, intensive care unit.

**Figure 5:**
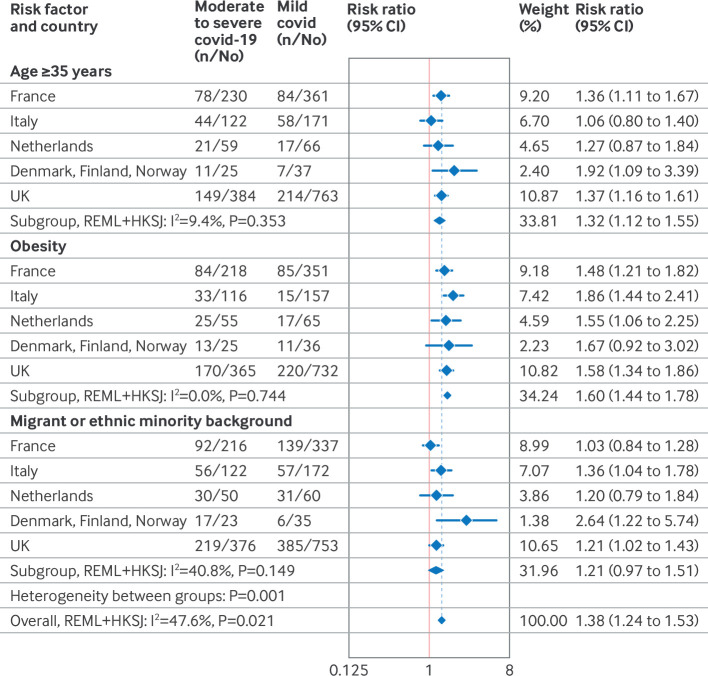
Crude risk ratio for moderate to severe covid-19 disease in pregnant women admitted to hospital for covid-19, stratified by age, obesity, and migrant or ethnic minority background, from 1 March to 31 December 2020, for each country. Non-European migrant background was defined as the mother's country of birth outside of Europe in all countries except the UK. In the UK, minority ethnic background was defined as Black, Asian, mixed, or other ethnic backgrounds. Random effects maximum likelihood model (REML) was used with Hartung-Knapp-Sidik-Jonkman (HKSJ) variance estimator. CI=confidence interval

Table 1 shows that the distribution of maternal characteristics varied across countries. In meta-analyses stratified by age, obesity, and migrant or ethnic minority background at country level, we found an increased risk of moderate to severe disease in women aged ≥35 years compared with women aged <35 years (pooled risk ratio 1.32, 95% CI 1.12 to 1.55, I^2^=9.4%, τ^2^<0.001), in women with obesity compared with women who were not obese (1.60, 1.44 to 1.78, I^2^=0%, τ^2^<0.001), and for women from a non-European migrant or ethnic minority background compared with women from a white (majority) or European born background (1.21, 95% CI 0.97 to 1.51, I^2^=40.8, τ^2^=0.003) (figure 5). Sensitivity analyses of risk ratios with adjustment for potential confounders based on larger datasets from France, Italy, and the UK showed minimal changes (to the second decimal) in adjusted risk ratios.

### Medical treatment

Table 3 outlines the main categories of medical treatment given alone or in combination to women in the covid-19 admission group by severity of covid-19 disease. Online supplemental table 5 gives more specific details. Information about corticosteroids for maternal indications was not available for Italy and Sweden.

**Table 3:** Specific medical treatments for covid-19 in pregnant women in the covid-19 admission group, by severity category and country, from 1 March to 31 December 2020

Country or region and severity of infection*	No of pregnant women	No (%) of women who received medical treatment for covid-19			
		Antiviral agents	Hydroxychloroquine	Corticosteroids for maternal indication	Low molecular weight heparin prophylaxis
Belgium:†					
Covid-19 admissions	68	1 (1.4)	15 (45.5)	9 (13.2)	28 (41.1)
France:‡					
Moderate to severe	230	29 (12.8)	16 (7.1)	77 (33.8)	174 (76.7)
Mild	361	4 (1.1)	3 (0.8)	7 (1.9)	131 (36.9)
Italy:					
Moderate to severe	122	53 (43.4)	58 (47.5)	NA	77 (63.1)
Mild	172	19 (11.2)	31 (18.2)	NA	91 (53.5)
Netherlands:‡					
Moderate to severe	59	4 (6.8)	6 (10.2)	2 (3.5)	NA
Mild	66	0	0	0	NA
Denmark, Finland, Norway:					
Moderate to severe	25	4 (16.0)	0	5 (27.8)	16 (88.9)
Mild	37	0	0	6 (16.2)	28 (75.7)
Sweden:†					
Admissions for covid-19	51	0	NA	NA	43
UK:§					
Moderate to severe	384	36 (9.4)	1 (0.3)	75 (19.5)	235 (95.1)
Mild	763	7 (0.9)	0	8 (1.0)	169 (54.9)

Values are number or number (%) by row (country).

Medical treatment for covid-19 could be given alone or in combination.

* Moderate to severe covid-19 was defined as maternal death, admission to an intensive care unit, or respiratory support with oxygen supplementation, high flow nasal oxygen or continuous positive airway pressure, mechanical ventilation, or extracorporeal membrane circulation.

† Data about some modalities of respiratory support were lacking from Belgium and Sweden.

‡ Information about respiratory support to women admitted to hospital for covid-19 was missing for 10 women in France and two women in the Netherlands.

§ Information about low molecular weight heparin prophylaxis was missing for 137 women in the UK, and percentages were calculated based on known information.

NA, not available.

**Figure 6:**
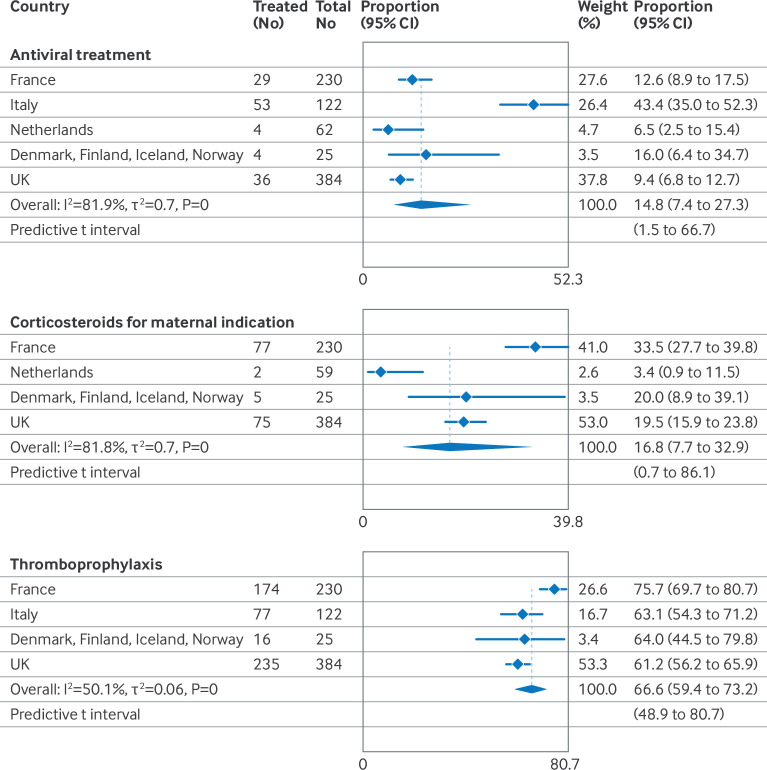
Proportion of pregnant women with moderate to severe covid-19 who were treated with antiviral medicines, corticosteroids for maternal indication, or thromboprophylaxis. Information about corticosteroids for maternal indication was not available from Italy, and information about thromboprophylaxis was not available from the Netherlands. CI=confidence interval

Prophylactic anticoagulation with low molecular weight heparin (thromboprophylaxis) was the most frequent medical treatment. Hydroxychloroquine was most frequently used in Italy and some Belgian regions but rarely in the other countries. Even among women with moderate to severe covid-19, few received other specific medical treatments for covid-19, with notable variations across countries (figure 6). Information about oxygen supplementation was not available in Belgium, and in Sweden data on respiratory support other than mechanical ventilation or extracorporeal membrane oxygenation, were not available. In countries where information about all modalities of respiratory support was available, 2219 women had moderate to severe covid-19; about one in six women received corticosteroids for a maternal indication (pooled proportion 16.8%, 95% CI 7.7% to 32.9%, I^2^=81.8, τ^2^=0.7), one in seven received antiviral treatment (14.8%, 7.4% to 27.3%, I^2^=81.9, τ^2^=0.7), and two of three women received thromboprophylaxis (66.6%, 59.4% to 73.2%, I^2^=50.1, τ^2^=0.06).

### Maternal secondary outcomes

Information about birth was available for 2214 (96.5%) women out of the 2350 covid-19 admissions. Median gestational age at birth for women in the covid-19 admission group was 38-39 weeks in all countries. Nevertheless, preterm birth before 37 weeks' gestational age was more common among women with moderate to severe covid-19, ranging from 26.7% in the Netherlands to 34.5% in the UK, noting that information about birth was missing for some women discharged with ongoing pregnancies in the Netherlands and Italy. Online supplemental table 6 has more details about preterm birth and mode of delivery by severity of covid-19. The pooled proportion of preterm birth before 34 weeks' gestation was 16.7% (95% CI 14.3% to 19.4%, τ^2^<0.001, prediction interval 12.9 to 21.2) in women with moderate to severe infection and 3.3% (1.6% to 6.4%, τ^2^=0.2, prediction interval 0.9 to 11.1) in women with mild infection.

Caesarean births included all indications, also indications not related to covid-19 disease. The pooled proportion of caesarean births was 52.9% (95% CI 46.3% to 59.5%, I^2^=42.2, τ^2^=0.05, prediction interval 41.6 to 64.0) in women with moderate to severe covid-19, and 31.6% (24.5% to 39.7%, I^2^=68.9, τ^2^=0.12, prediction interval 18.8 to 47.8) in women in the covid-19 admission group who did not need respiratory support.

### Perinatal secondary outcomes

Information was available for 2283 infants born after 22 weeks' gestational age. Stillbirths and neonatal deaths were rare, with no events in many countries (online supplemental table 7). Half of the stillbirths were reported for women with moderate to severe covid-19 (13/24), but with potential bias because some missing information about births to women discharged while still pregnant in Italy and the Netherlands. Among infants with available outcome information, admission to a neonatal ward was more common in infants born to women with moderate to severe infection (32.8%, 95% CI 25.9% to 40.5%, τ^2^=0.09, prediction interval 20.9 to 47.4 *v* 13.3%, 11.7% to 15.3%, τ^2^<0.0001, prediction interval 10.6 to 16.5, for admission to the neonatal ward for infants born to women with mild infection).

## Discussion

### Principal findings

This study showed large variations in the incidence of hospital admissions for covid-19 disease in pregnant women in 10 European countries. The incidence of admissions ranged from no admissions to almost 2 per 1000 maternities during the study period, with the lowest estimates in four Nordic countries (Denmark, Finland, Iceland, and Norway) and the highest in Sweden, France, and the UK. Few of the pregnant women with moderate to severe disease received medical treatment specific to SARS-CoV-2 infection, such as antiviral medicines or corticosteroids.

### Strengths and limitations of this study

Whilst the first published studies of pregnant women with covid-19 were small, the following emphasis of larger studies and greater numbers of individuals had the potential to overlook important lessons from countries with a low risk of hospital admissions for covid-19 disease. The population based approach in our study reduced the risk of several biases, such as selection bias, and also of publication bias, where studies from smaller populations would be less likely to be published. Aligned definitions of cases (ie, exposure to the virus and cause of admission),outcomes, and covariates added to the comparability of the national results, including the active surveillance to identify all maternal deaths.^37^,^39^

Although pregnant women might need healthcare related to the pregnancy, many previous studies have not clearly discriminated between hospital admissions for pregnancy related healthcare and admissions for covid-19 disease.^15^ The availability of clinical information about the cause of hospital admission or symptoms was essential for better categorisation and to avoid incorrectly attributing severe pregnancy outcomes in asymptomatic women with obstetric complications to infection with the SARS-CoV-2 virus.^40^ All of the countries participating in INOSS had policies to test pregnant women with symptoms of covid-19 disease during the study period (online supplemental figure 1), and we assumed that public health interventions to reduce personal movement did not affect access to necessary healthcare. However, the testing strategies would impact the detection of asymptomatic infection among pregnant women in need of pregnancy related healthcare in labour or obstetric emergencies.^41^ Therefore, a strength of our study was that we could evaluate risk depending on symptom status. Stratification of analyses by country accounted to some extent for differences in the risk of infection, social support, and population density, as well as the characteristics of the study population and national care practices, such as baseline caesarean birth rates.

Strict national regulations in some countries following the European General Data Protection Regulation (published in 2016) prevented international sharing of individual health information, thus requiring a two stage approach to some analyses, with national analyses performed with the teams' preferred statistical analysis programme. Regional data were available from France (60% of national births) and Sweden (31% of national births), indicating that more caution is needed with regards to national generalisability and selection bias in these countries. All countries and regions were likely to identify pregnant women in need of hospital care, however, and all countries had systems in place to ascertain complete reporting.

Our study had several limitations. Firstly, we could not describe the incidence of SARS-CoV-2 infection in pregnant women who were not admitted to hospital. Secondly, severe perinatal outcomes were rare, and were not restricted to covid-19 disease as the main cause, even among women with moderate to severe disease. Thirdly, for the available covariates, age was categorised in our study based on published research and national results. The cut-off point at age 35 years was arbitrary and age would be better modelled as a continuous covariate in future studies. Although non-European migrant background was not equivalent to ethnic minority background, we had to adapt to different practices for recording and reporting this information in different countries.^42^ Lastly, limited information was available for some risk factors that could be important in the interpretation of the results, such as socioeconomic status.

### Comparison with other studies

Although few studies have reported population based estimates for the incidence of hospital admissions for covid-19 disease in the pregnant population, other population based sources have described a similar distribution of incidence of severe covid-19 and deaths related to covid-19 disease in different countries. Based on data from the Organisation for Economic Co-operation and Development and the European Centre for Disease Control, Denmark, Finland, and Norway were among the countries with the lowest number of deaths per million inhabitants in 2020, and Sweden, France, UK, Italy, and Belgium were among the countries with the highest mortality.^8^ Sweden, France, UK, Italy, and Belgium also had the highest estimates for the total population infected with the SARS-CoV-2 virus.^43^ The Oxford stringency index was developed as a summary description of government responses to covid-19, and a high early index was associated with reduced mortality.^44^ According to the government response tracker used for the stringency index, Italy was the first to implement measures resulting in a stringency index >50 on 24 February 2020, and the UK and Sweden were the last to implement such measures, on 21 March and 26 March 2020, respectively.^44^ Denmark, Finland, Iceland, and Norway reached a stringency index of >50 on 13-17 March 2020.

When interpreting differences in the incidence of hospital admissions related to covid-19 disease, the distribution of risk factors for covid-19 in the pregnant population must also be considered. Women aged ≥35 years, with obesity, or from a non-European migrant or ethnic minority background were at greater risk for moderate to severe covid-19 in our study. The risk ratio estimates were lower than the pooled odds ratios reported in the WHO systematic review, as expected when moderate to severe covid-19 was common among the included women.^15^ Comparison with studies included in the WHO review is difficult because the definitions of causes of admissions to hospital or an intensive care unit were not always clear.

The sociodemographic characteristics and risk factors among women in the source populations in participating countries varied to some extent according to European perinatal surveillance and official statistics.^45^,^48^ People from a migrant or ethnic minority background have been shown to be over-represented in hospital admissions for covid-19,^42^ and this trend was also seen in our study for most of the countries. However, differences in age, obesity, and non-European migrant or ethnic minority background in pregnant populations in each country are unlikely to explain the observed variation in incidence of hospital admissions for covid-19 across the countries. The variation in the prevalence of risk factors in different countries indicates the need for careful consideration of factors such as baseline risk in pooling and analyses of multi-country studies.^49^

Population density, social security system, and social inequalities might also have a role in viral transmission and risk of disease. Although the Organisation for Economic Co-operation and Development inequality indices (Gini index) for the Nordic countries are among the lowest in Europe, social inequality indicators or European population density studies do not seem to fully explain the observed difference in incidence.^50^,^52^

During the first and second waves of the pandemic, pregnant women with moderate to severe covid-19 did not receive medical treatment specific to covid-19 disease, even when there were few safety concerns and after the RECOVERY trial indicated a reduction in severe morbidity and death with early corticosteroid treatment in adults needing respiratory support.^53^ The apparent under-treatment aligns with previous experiences with epidemic threats, with persisting bias for vaccine development and vaccination,^16^ medical treatment,^54^ and also in the development of clinical guidelines.^55^

Maternal and perinatal outcomes were secondary outcomes in our study, the observation time was relatively short, and information about birth and infant outcomes was not complete in some countries for women who were discharged with an ongoing pregnancy and gave birth in a different hospital. However, the available information about outcomes from the index admission suggest a higher risk of preterm birth, caesarean birth, and admission to a neonatal ward in women with moderate to severe infection. Although our study cannot offer conclusive evidence, these results indicate the merit of nuanced analyses, taking severity of covid-19 into account when assessing maternal and perinatal outcomes in future studies.

In summary, the highest incidence of hospital admissions for covid-19 disease was seen in countries with a high burden of disease and was unlikely to reflect higher thresholds for hospital admission or lower capacity for admission to the intensive care unit in countries with a low incidence. The observed differences were unlikely to be explained only by differences in the distribution of risk factors in the pregnant source populations, or by other societal factors, such as population density and social inequality. The surveillance was based on a joint protocol and aligned definitions of cases, outcomes, and covariates. Although minor adaptations had to be made in different countries, methodological differences are unlikely to explain the observed difference in incidence of hospital admissions for covid-19. Our study indicates that public health policies might have had a largely unacknowledged role in protecting pregnant women from severe disease. The considerable under-treatment of pregnant women raises concerns for future outbreaks, epidemics, and pandemics.

### Study implications for research and practice

Although our study was conducted in high income countries, the meta-analysis from Europe might be relevant for future analysis and interpretation of multi-country studies, particularly for differences in incidence potentially related to health policies. Previous studies, such as the multinational cohort INTERCOVID study, reported an increased risk of maternal and neonatal mortality in low and middle income settings, but information about the source populations were not available, and the studies were hospital based.^56^ The INOSS surveillance model has been successfully applied in South Africa and India. The South African Obstetric Surveillance system reported high fatality rates among pregnant women admitted to hospital for covid-19.^57^ In India, lockdowns were associated with reduced access to essential reproductive healthcare and an increase in severe maternal morbidity and case fatality from complications related to pregnancy.^58^

The meta-analysis indicates that public health measures aimed at reducing deaths and limiting viral transmission in the society might also protect pregnant women.^59^ Combining data from several countries at a European level allowed insights into national trends, with the potential to inform national policies on surveillance of incidence and severe outcomes, medical treatment of pregnant women, and personal protection by vaccination.^60^

The difference in incidence indicates that national policies might have had a substantial role on the risk of hospital admissions for covid-19. If this knowledge had been available before the second wave of the pandemic in 2020 and before the alpha and delta variant dominant periods of the SARS-CoV-2 virus, severe maternal disease and deaths might have been prevented. Several INOSS countries used preliminary results from the national surveillance to inform national guidelines for the care of pregnant women,^61^,^63^ but national guidelines continued to vary widely during the pandemic.^55^ In future epidemics or pandemics, rapid and robust population based surveillance might also inform international treatment guidelines for pregnant women.

Our study identified several challenges in providing timely, robust information about the effect of the pandemic on pregnant women. Online supplemental file 1 outlines the surveillance models implemented in each country. Slow approval and data collection processes hampered the response, and organisational challenges, such as lack of funding for data collection and analysis, and lack of capacity and staff, were further barriers to rapid analysis and dissemination. Similarly, a survey of perinatal health surveillance in Europe during the pandemic reported large variations in the availability of routine surveillance data for perinatal health, and in some countries perinatal health surveillance was delayed as a result of core staff being allocated to other pandemic related tasks.^64^

Several INOSS countries concluded their surveillance early or late in the alpha variant dominant period of the SARS-CoV-2 virus in spring 2021 because of lack of capacity and funding. The INOSS countries with ongoing surveillance when the delta variant of the SARS-CoV-2 virus was the dominant strain could rapidly compile and share results about severe covid-19 and vaccination status, showing that most women admitted to intensive care units had not been vaccinated.^65^ Some countries followed up on vaccination status and outcomes during the period when the omicron variant of the SARS-CoV-2 virus was the dominant strain.^19 60^

Covid-19 went on to become the most common cause of maternal deaths reported in the Confidential Enquiry for the triennium 2019-21 in the UK, and most of the women who died were not vaccinated.^5^ Robust and reliable information about the consequences of disease is essential for pregnant women and their partners in seeking care and deciding about personal protection, such as vaccination.^10^ Consequently, European investment in robust surveillance systems is urgently needed to provide timely and relevant information to authorities and pregnant women and their partners.

### Future research

Further confidential enquiries into maternal deaths and severe disease could provide important learning for clinical care and emergency preparedness. Future work should aim to develop robust surveillance and systems to rapidly compare findings in different countries. With combined efforts, we can ensure that pregnant women benefit from at least the same level of prevention, detection, and care as the general populations during health emergencies.^66^

### Conclusions

Population based surveillance in 10 European countries during the first 10 months of the covid-19 pandemic showed large variations in the risk of hospital admissions for covid-19 in pregnant women. This finding indicates that national public health policies likely had a substantial and previously unrecognised role in protecting pregnant women. Few pregnant women with moderate to severe covid-19 disease were given specific medical treatment for SARS-CoV-2 infection, even when no or minor safety concerns existed. Lessons for future pandemics include the importance of rapid, robust surveillance systems for maternal and perinatal health, and using pregnant women early in the development and testing of medicines and vaccines for public health emergencies.

## Supplementary material

10.1136/bmjmed-2023-000733 online supplemental file 1

10.1136/bmjmed-2023-000733 Uncited online supplemental file 1

10.1136/bmjmed-2023-000733 Uncited online supplemental file 2

## Data Availability

No data are available.
