# Supplementary material for: Pregnant women admitted to hospital with covid-19 in 10 European countries: individual patient data meta-analysis of population based cohorts in International Obstetric Survey Systems
Source: BMJ Med. 2024 Dec 12;3(1):e000733. doi: 10.1136/bmjmed-2023-000733 (PMC12164325; doi:10.1136/bmjmed-2023-000733)
Supplement: online supplemental file 1 [file bmjmed-3-1-s001.pdf]

Supplementary files

Table of content

1.Data sources and data quality verification 2

1.2 Belgium 3

1.3 France 3

1.3 Italy 3

1.4 The Netherlands 3

1.5 Nordic countries (NOSS), Denmark, Finland, Iceland, Norway and Swedish regions 3

1.6 United Kingdom 4

2.Testing strategies 5

3.Total maternities, covid-19 admissions and non-covid admissions 6

4.Pre-pregnancy chronic disease and pregnancy-related disease amongst women admitted due to covid-19 7

5.Meta-analysis of incidence using a hierarchical model with iterative process (“metapreg”) and a model with Freeman-Tukey double arcsine transformation(“metaprop”) 8

6.Covid-specific medical treatment to pregnant women in the covid-19 admission group 9

7.Maternal secondary outcomes 10

7.Perinatal secondary outcomes 11

8. References 12

1.Data sources and data quality verification

Supplementary table 1. Overview of participating countries and source populations

	B.OSS	France	ItOSS	NethOSS	Nordic NOSS					UKOSS
Country	Belgium	France	Italy	The Netherlands	Denmark	Finland	Iceland	Norway	Sweden	United Kingdom
Country abbreviation	Be	Fr	It	Nl	Dk	Fi	Is	No	Se	UK
Geographical area	National	6 regions (60% of national)	National	National	National	National	National	National	3 university hospital 3 regions, (31% of national)	National
No. women giving birth per year[2]	124K	480K (60% of national total 705K)	420K	170-185K	60K	46K	5K	55K[3]	36K (±31% of national total 115K)	650-750K
Date first covid-19 pregnancy recruited	01/03/2020	01/03/2020	25/02/2020	01/03/2020	01/03/2020	**01/03/2020**	**01/03/2020**	24/03/2020	01/03/2020	01/03/2020

[1] By population-based in this context, we mean is there an effort to include every eligible pregnancy in the geographical area
[2] Approximate no. annual pregnancies in the area covered by the survey cohort.
[3] The Medical Birth Registry of Norway receives mandatory reports for all abortions/births >12 weeks gestational age, cases prior to 12 weeks gestational age are included if reported.

The INOSS countries collected national or regional case reports for pregnant women with SARS-COV-2 infection admitted to hospital. The case report form was uniform but allowed for minor adaptation to national requirements, such as classification of minority or migrant background. Information in the case report form was retrieved from prospectively entered information in the patient records. Some member countries performed quality control for completeness using other sources such as national health registries. Data quality verification, for instance with national covid-19 infection registries, was not possible for all countries, but if available this source for data quality verification was also used. Country specific information is outlined below.

1.2 Belgium

B.OSS collected information about admission to hospital for women with covid-19 infection in pregnancy and up to 42 days postpartum. Cases were notified by reporting clinicians at each hospital. Enrolment was closed at the end of February 2021. Enrolled women were followed till the end of pregnancy. National perinatal statistics for 2020 were available in December 2021 as denominator data. BOSS data could not be linked to hospital discharge databases because it was not possible to obtain agreement with the Committee of Security Information. BOSS data was linked to perinatal registries, and denominator perinatal data were available nationwide, but separately for Flanders, Brussels and Wallonia.

1.3 France

Women with SARS-CoV-2 infection during pregnancy and up to 7 days postpartum, hospitalised or not, were prospectively identified in a local registry in all maternity units of six French regions and included in the COROPREG study (NCT04463758).[1] They were followed until the end of pregnancy. Linkage with the French National Health Data System (SNDS) is planned. Information about maternal deaths were cross-checked with the national maternal death surveillance (ENCMM). The number of births for denominators were extracted from the national hospital discharge database.

1.3 Italy

ItOSS sent out weekly notification reminders to identify new cases and contacted clinicians by email and telephone in case of zero reporting. The completeness of the data collection forms was verified monthly, and clinicians were requested to complete missing data. Maternal deaths were cross-checked with the maternal mortality surveillance system. For the current this data check has been completed.[2 3] During the study period in 2020, 327 pregnancies were ongoing at time of hospital discharge. Follow-up information after discharge about secondary outcomes was available for 168/327 (51.4%) women admitted due to covid-19 or symptomatic and for 93/122 (76.2%) of women with moderate to severe covid-19. Denominator data were nationwide and from the medical birth registry.

1.4 The Netherlands

The Netherlands Obstetric Surveillance System (NethOSS) works under the umbrella of the Perined database. Perined was established in 1971 as the nationwide perinatal database. It contains 98-99% of all pregnancy and birth outcomes in the Netherlands. National denominator data from 2020 were available. NethOSS sent out weekly reminders to reporting clinicians with a reporting link specific for each clinician.[4] It was not possible to link NethOSS to the national registry for COVID-testing (RIVM surveillance registry). The NethOSS team could verify the reported information and if neccessary contact the reporting clinician if information was missing in the primary report.

1.5 Nordic countries (NOSS), Denmark, Finland, Iceland, Norway and Swedish regions

National data were collected in all Nordic countries except Sweden where women from the following areas were included: the University Hospitals at Karolinska, Sahlgrenska and Lund-Malmö, and the regions of Halland, Dalarna and Vestmannland, the areas represent approximately 31% of annual maternities in Sweden.

NOSS Denmark sent bimonthly reminders to clinicians during the study period. NOSS Denmark was linked to the National Microbiology database and the Danish Patient Registry to verify complete reporting, and missing cases were entered retrospectively.(https://pubmed.ncbi.nlm.nih.gov/36695168/)
NOSS Finland linked the Finnish Medical Birth Register to the National Infectious Disease Register and the Hospital Discharge Register and could retrieve clinical information from patient records.
NOSS Iceland linked the Infectious Disease Registry and the National Discharge Database (SAGA).
The Medical Birth Registry of Norway sent bimonthly reminders to clinicians during the study period and asked clinicians to confirm the number of new cases or confirm no new cases to report. Due to legal regulation of registry data use and delays in linkages, it was not possible to link the Medical Birth Registry of Norway with other health registries in Norway for the current study.

National Medical Birth Registries:

Denmark (https://www.dst.dk/en/Statistik/emner/borgere/befolkning/foedsler),
Finland: total number of births from The Finnish institute of Health and Welfare (https://thl.fi/en/web/thlfi-en/statistics-and-data/statistics-by-topic/database-reporting),
Iceland: total number of births from Statistics Iceland (https://www.statice.is/statistics/population/births-and-deaths/births/),
Norway: total number of births from The Medical Birth Registry of Norway (http://statistikkbank.fhi.no/mfr/)

All the Nordic countries could verify the reported information or retrieve missing information from medical records, further details have been published.[5 6] National perinatal statistics for 2020 were published in April 2021 in Norway, and perinatal statistics for 2020 were published between October 2021 and January 2022 for the other Nordic countries, and in a joint report by The Nordic medical Birth Registries (NOMBIR) collaboration.[7]

Due to national GDPR regulations numbers less than 3 cannot be reported by country, consequently national datasets were merged in the meta-analysis, further details are given in the relevant table footnotes.

1.6 United Kingdom

UK Obstetric Surveillance System (UKOSS) reporters were requested to report cases daily via a live reporting link and monthly notification reminders were sent to reporting clinicians at all hospitals with an obstetric unit to identify new cases. Following the notification of a case clinicians were reminded to complete the data collection form (https://www.npeu.ox.ac.uk/ukoss/completed-surveillance/covid-19-in-pregnancy) Information about maternal or neonatal deaths was cross-checked with the national maternal and perinatal death surveillance, MBRRACE-UK (https://www.npeu.ox.ac.uk/mbrrace-uk). UKOSS data could not be linked to other national health registries. For the data used in this study missing information about covariates were <5% of cases.

2.Testing strategies

In all countries symptomatic pregnant women who needed hospital admission would have been tested throughout the study period. Some countries implemented screening early in the period; testing of all pregnant women who needed hospital admission. Screening would lead to more asymptomatic women being included, while admitted for labor or other pregnancy related health care. Supplementary figure 1 below illustrates the different strategies in the participating countries during the study period. Belgium did not have a national strategy and testing policies differed across regions. Similarly, Sweden introduced screening early in some regions but not in all participating areas.

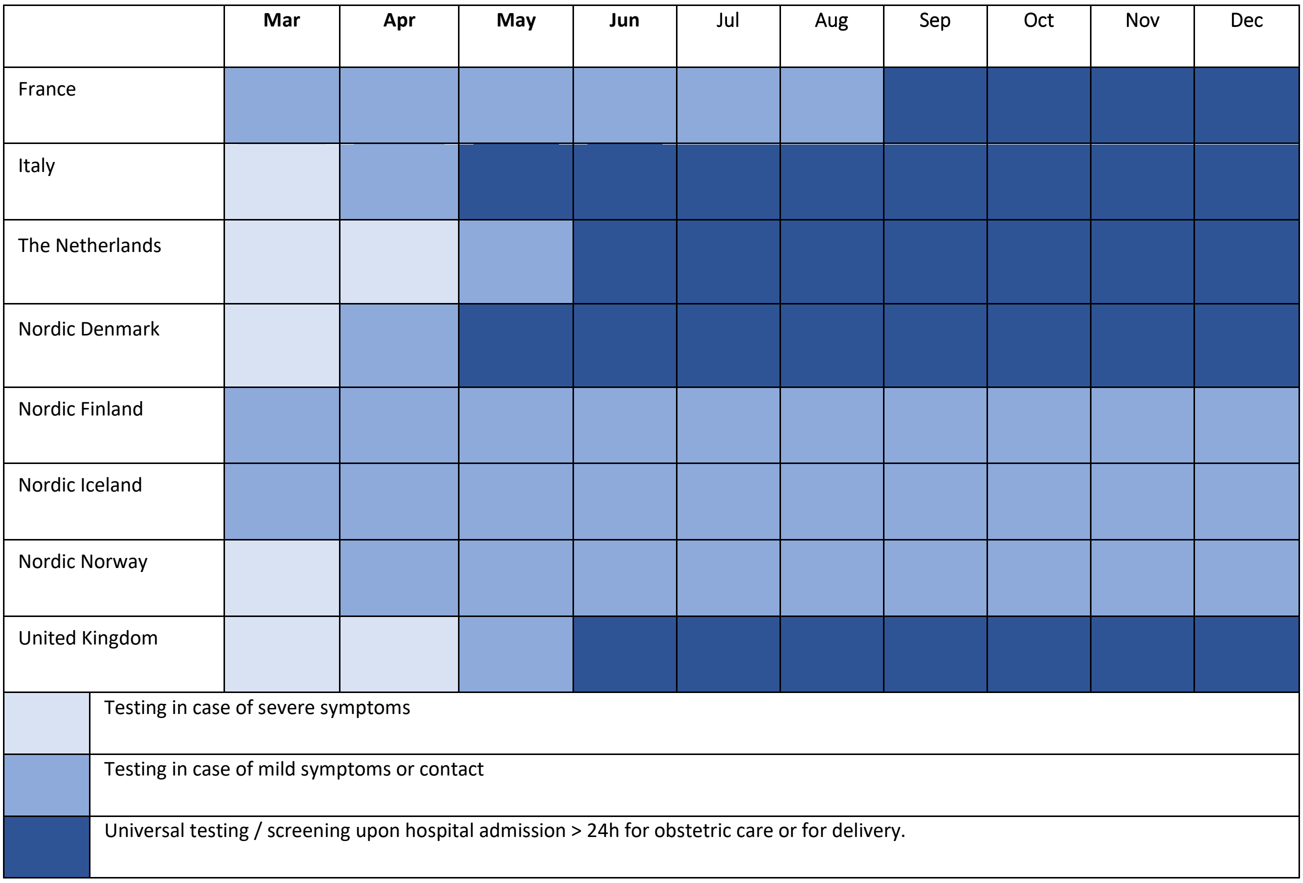

Supplementary Figure 1. National SARS-CoV-2 testing strategies by month and country.

3.Total maternities, covid-19 admissions and non-covid admissions

Supplementary Table 2. Total number of maternities, covid-19 admissions and non-covid admissions per 1000 maternities, by country, 1 March to 31 December 2020

Country	Maternities	Covid-19 admissions	Non-covid admissions
Total	1.7 million	2 350	6 653
Belgium<br>n (per 1000)	95463	68 (0.7)	641 (6.7)
France*<br>n (per 1000)	361500	601 (1.7)	979 (2.5)
Italy<br>n (per 1000)	335922	294 (0.9)	2020 (6.0)
The Netherlands<br>n (per 1000)	141691	127 (0.9)	242 (1.7)
Nordic Denmark<br>n (per 1000)	51050	36 (0.7)	91 (1.78)
Nordic Finland<br>n (per 1000)	39616	16 (0.4)	13 (0.3)
Nordic Island<br>n (per 1000)	3750	0	<3 (0.5)†
Nordic Norway<br>n (per 1000)	47431	10 (0.2)	15 (0.3)
Nordic Sweden*<br>n (per 1000)	36626	51 (1.4)	182 (45.0)
United Kingdom<br>n (per 1000)	608050	1147 (1.9)	2468 (4.0)

*Regional surveillance, the total maternities during the study period were based on numbers from participating hospitals.
† Due to national data protection regulations, national counts less than three are not reported for the Nordic countries.

4.Pre-pregnancy chronic disease and pregnancy-related disease amongst women admitted due to covid-19.

Supplementary Table 3. Covid-19 severity category and pre-pregnancy chronic disease and pregnancy-related disease among pregnant women admitted due to covid-19 or with symptoms, by country, 1 March to 31 December 2020

Country	Severity*	No of pregnant women†	Hypertension prior to pregnancy		Diabetes prior to pregnancy		Gestational diabetes		Preeclampsia	
			yes	no	yes	no	yes	no	yes	no
Belgium‡ n (%)	covid-19 admissions	68	2 (2.9)	66 (97.1)	0	68	12 (17.6)	56 (82.4)	1 (1.5)	67 (98.5)
France§ n (%)	Moderate to severe	230	11 (4.8)	218 (95.2)	13 (5.7)	216 (94.3)	51 (22.5)	176 (77.5)	12 (5.3)	215 (94.7)
	Mild	361	13 (3.6)	348 (96.4)	10 (2.8)	351 (97.2)	82 (22.8)	277 (77.2)	13 (3.6)	346 (96.4)
Italy n (%)	Moderate to severe	122	7 (5.9)	111 (94.1)	4 (3.4)	114 (96.6)	11 (9.6)	103 (90.4)	2 (1.8)	112 (98.2)
	Mild	172	0	164	3 (1.8)	161 (98.2)	16 (10.1)	142 (89.9)	0	158
The Netherlands§ n (%)	Moderate to severe	59	1 (1.7)	58 (98.3)	2 (3.4)	57 (96.6)	4 (6.8)	55 (93.2)	0	59
	Mild	66	0	66 (100)	1 (1.5)	65 (95.5)	0	66 (100)	4 (6.1)	62 (93.9)
Nordic Dk, Fi, No n (%)	Moderate to severe	25	1 (4.0)	24 (96.0)	1 (4.0)	24 (96.0)	2 (11.1)	16 (88.9)	2 (8.0))	23 (92.0)
	Mild	37	0	50 (100)	1 (2.5)	49 (98.0)	11 (22.0)	39 (78.0)	1 (2.0)	49 (98.0)
Nordic Se‡ n (%)	covid-19 admission	51	0	51 (100)	1 (2.0)	50 (98)	10 (19.6)	4 (80.4)	2 (3.9)	49 (96.1)
United Kingdom n (%)	Moderate to severe	384	22 (5.7)	362 (94.3)	13 (3.4)	371 (96.6)	58 (15.1)	326 (84.9)	3 (0.8)	381 (99.2)
	Mild	763	14 (1.8)	749 (98.2)	11 (1.4)	752 (98.6)	77 (10.1)	686 (89.9)	10 (1.3)	753 (98.7)

Abbreviations for the Nordic countries: Denmark (Dk), Finland (Fi), Iceland (Is), Norway (No), Sweden (Se). There were no covid-admissions in Iceland.

* Moderate to severe covid-19 was defined as maternal death, intensive care unit admission, or maximum respiratory support with either of the following: oxygen, high flow nasal oxygen or continuous positive airway pressure, mechanical ventilation or extracorporeal membrane oxygenation. Women with mild disease were admitted due to covid-19 or symptomatic but received only supportive therapies such as intravenous fluids and inhalations and not respiratory support.

† If the sum of categories of characteristics is less than the total this is due to missing information. Percentages are calculated based on cases with known information.

‡ Belgium and Sweden did not have detailed data on respiratory support with oxygen and could not discriminate between the severity categories among women admitted due to covid-19 in the current dataset.

§ Information about need for respiratory support was lacking for 10 women in France and for 2 women in the Netherlands.

5.Meta-analysis of incidence using a hierarchical model with iterative process ("metapreg") and a model with Freeman-Tukey double arcsine transformation("metaprop")

Supplementary Table 4. Incidence of admission due to covid-19 per 1000 maternities with the two different estimation methods for meta-analysis of incidence, by country, March to December 2020.

	Admissions due to covid using metapreg			Admissions due to covid-19 using metaprop			
Country	Incidence per 1000 materntities (95% CI])			Incidence per 1000 maternities (95% CI)			weight
Belgium	0.71	(0.56	0.90)	0.71	(0.56	0.90)	10.52
France	1.64	(1.52	1.78)	1.64	(1.52	1.78)	10.77
Italy	0.88	(0.78	0.98)	0.88	(0.78	0.98)	10.76
The Netherlands	0.90	(0.75	1.07)	0.90	(0.75	1.07)	10.63
Nordic Dk	0.71	(0.51	0.98)	0.71	(0.51	0.98)	10.24
Nordic Fi	0.40	(0.25	0.66)	0.40	(0.25	0.66)	10.08
Nordic Is	0.00	(0.00	1.02)	0.00	(0.00	1.02)	5.98
Nordic No	0.21	(0.11	0.39)	0.21	(0.11	0.39)	10.20
Nordic Se	1.39	(1.06	1.83)	1.39	(1.06	1.83)	10.02
United Kingdom	1.89	(1.78	2.00)	1.89	(1.78	2.00)	10.80
Overall	0.76	(0.49	1.18)	0.81	(0.52	1.17)	100.00
Prediction interval		(0.25	2.30)				

Test of heterogeneity

- LR Test: RE model vs FE model

	DF	Chisq	p	tau2	I^2
Overall	1.00	368.63	0.00	0.44	87.55

Test(s) of heterogeneity:

	Heterogeneity statistic	degrees of freedom	P	I^2**
	449.59	9	0.00	98.00%
Overall	449.59	9	0.00	98.00%

** I^2: the variation in ES attributable to heterogeneity)

Significance test(s) of ES=0

Admitted due to covid	z= 8.69	p = 0.00
Overall	z= 8.69	p = 0.00

Abbreviations: CI: Confidence Interval. The Nordic countries: Denmark (Dk), Finland (Fi), Iceland (Is), Norway (No), Sweden (Se).

6.Covid-specific medical treatment to pregnant women in the covid-19 admission group

Supplementary Table 5: Covid-19 specific medical treatment to pregnant women in the covid-19 admission group, by severity category and country, 1 March to 31 December 2020.

Country	Severity category*		Nr of pregnant women	Antiviral	Antibiotics	Hydroxy-chloroquine	Corticosteroids		Tocilizumab	Low molecular weight heparin	
							total	maternal indication		prophylaxis	treatment
Belgium†	covid-19 admissions	N (%)	68	1 (1.4)	22 (32.4)	15 (45.5)	19 (27.9)	9 (13.2)	0	28 (41.1)	1 (1.5)
France‡	Moderate to severe	N (%)	230	29 (12.8)	22 (9.7)	16 (7.1)	111 (48.3)	77 (33.8)	0	174 (76.7)	17 (7.5)
	Mild	N (%)	361	4 (1.1)	9 (2.5)	3 (0.8)	59 (16.3)	7 (1.9)	0	131 (36.9)	2 (0.6)
Italy	Moderate to severe	N (%)	122	53 (43.4)	82 (67.2)	58 (47.5)	na	na	0	77 (63.1)	0
	Mild	N (%)	172	19 (11.2)	57 (33.5)	31 (18.2)	na	na	0	91 (53.5)	0
The Netherlands‡	Moderate to severe	N (%)	59	4 (6.8)	22 (37.3)	6 (10.2)	22 (37.3)	2 (3.5)	0	na	na
	Mild	N (%)	66	0	21 (31.8)	0	3 (4.5)	0	0	na	na
Nordic Dk, Fi, No	Moderate to severe	N (%)	25	4 (16.0)	9 (36.0)	0	9 (36.0)	5 (27.8)	0	16 (88.9)	2 (11.1)
	Mild	N (%)	37	0	9 (24.3)	0	9 (24.3)	6 (16.2)	0	28 (75.7)	1 (2.7)
Nordic Se†	covid-19 admissions	N (%)	51	0	na	na	7	na	na	43	na
United Kingdom§	Moderate to severe	N (%)	384	36 (9.4)	175 (45.6)	1 (0.3)	183 (47.7)	75 (19.5)	4 (1.0)	235 (95.1)	0
	Mild	N (%)	763	7 (0.9)	145 (19.0)	0	81 (10.6)	8 (1.0)	0	169 (54.9)	1 (0.1)

The different categories of covid-19 directed medical treatment could be administered alone or in combination.
Abbreviations: Nordic countries: Denmark (Dk), Finland (Fi), Iceland (Is), Norway (No), Sweden (Se). na=not available
* Moderate to severe covid-19 was defined as maternal death, intensive care unit admission, or any of the following respiratory support; oxygen supplementation, high flow nasal oxygen or continuous positive airway pressure, mechanical ventilation or extracorporeal membrane circulation.
† Belgium and Sweden lacked data about some modalities of respiratory support
‡ Information about respiratory support among covid-19 admissions was missing for 10 women in France and 2 women in the Netherlands.
§ Information about low molecular weight heparin prophylaxis was missing for 137 women in the UK, percentage calculated based on known information

7.Maternal secondary outcomes

Supplementary Table 6. Preterm birth and mode of birth among pregnant women in the covid-19 admission group, by covid-19 severity and country, March 1 to December 31 2020.

Country/ region	Severity	Covid-19 admissions	Total women with birth information available	Preterm birth			Mode of birth	
				Total births < 37 gestational weeks	<34 gestational weeks	34-36 gestational weeks	Vaginal birth	Caesarean birth‡
Belgium n (%)*	Admitted due to covid-19	68	68 (100.0)	9 (13.2)	na	na	50(73.5)	18(26.5)
France n (%)*	Moderate to severe	230	228 (99.1)	77 (33.6)	38 (16.6)	39 (17.0)	127 (55.7)	101 (44.3)
	Mild	361	357 (98.9)	37 (10.3)	15 (4.2)	22 (6.1)	248 (69.5)	109 (30.5)
Italy, n (%)*	Moderate to severe	122	93 (76.2)	29 (31.1)	16 (17.2)	13 (14.0)	40 (44.0)	51 (56.0)
	Mild	172	128 (74.4)	9 (7.0)	1 (0.8)	8 (6.3)	88 (68.7)	40 (31.3)
The Netherlands n (%)*	Moderate to severe	62	45 (72.6)	12 (26.7)	9 (20.0)	3 (6.7)	24 (53.3)	21 (46.7)
	Mild	63	56 (88.9)	8 (14.3)	4 (7.1)	4 (7.1)	38 (67.9)	15 (26.8)
Nordic Dk, Fi, No n (%)*	Moderate to severe	25	24† (100)	7 (29.2)	4 (16.7)	3 (12.5)	9 (37.5)	15 (66.7)
	Mild	37	36 (97.3)	1 (2.7)	0	1	28 (75.7)	11 (25.7)
Nordic Se n (%)*	Admitted due to covid-19	51	50	9 (20.9) §	na	na	30 (61.2)	19 (38.8)
UK n (%)*	Moderate to severe	384	377† (98.2)	130 (34.5)	70 (18.4)	60 (15.8)	153 (40.6)	224 (59.4)
	Mild	763	728 (95.4)	106 (14.6)	36 (4.7)	70 (9.2)	420 (56.2)	328 (43.8)

Abbreviations for the Nordic countries: Denmark (Dk), Finland (Fi), Iceland (Is), Norway (No), Sweden (Se).

* Percentages are presented out of cases with known information

† One woman in the Nordic countries and in the UK was included postpartum

‡ All cesarean births, also other indications than covid-19.

§ Information about gestational age at birth was missing for seven women in Sweden, and mode of birth was missing for one woman.

7.Perinatal secondary outcomes

Supplementary Table 7: Neonatal outcomes after birth to SARS-Cov-2 positive pregnant women who had been admitted to hospital due to covid-19, between March 2020 and December 2020

Country/ region n (%)	Severity	Total births after covid-19 admission with information*	Live births	Stillbirth	Neonatal death	Neonatal ward admission	Birthweight <10 percentile for GA and sex, singleton
Belgium n (%)	Admitted due to covid-19	71	71 (100)	0	1 (1.4)	16 (22.5)	
France n (%)	Moderate to severe	236	233 (98.7)	3 (1.3)	2 (0.9)	96 (41.2)	17 (8.0)
	Mild	380	374 (98.4)	6 (1.6)	2 (0.5)	56 (15.0)	44 (12.8)
Italy n (%)	Moderate to severe	95	91 (95.8)	4 (4.2)	0	20 (21.1)	5 (5.6)
	Mild	131	130 (99.2)	1 (0.8)	0	11 (8.5)	11 (8.8)
The Netherlands n (%)	Moderate to severe	45	45 (100)	0	0	12 (26.7)	1 (2.2)
	Mild	56	56 (100)	0	0	10 (17.9)	2 (3.6)
Nordic Dk, Fi, No, n (%)	Moderate to severe	24	24 (100)	0	0	11 (45.8)	1 (4.2)
	Mild	36	36 (100)	0	0	6 (16.7)	1 (2.8)
Sweden n (%)	Admitted due to covid-19	52	52	0	0?	9 (17.3)	2 (3.8)
United Kingdom, n (%)	Moderate to severe	390	384 (98.4)	6 (1.6)	2 (0.5)	130 (33.9)	55 (15.7)
	Mild	767	763 (99.5)	4 (0.5)	2 (0.3)	99 (13.0)	121 (16.8)

Abbreviations for the Nordic countries: Denmark (Dk), Finland (Fi), Iceland (Is), Norway (No), Sweden (Se).

* Two women (one woman each in the Nordic countries and in the UK) were included after birth.

8. References

1. Deneux-Tharaux C. Covid-19 in Pregnancy: a French Population-based Cohort of Women and Newborns. ClinicalTrials.gov, 2020.
2. Donati S, Corsi E, Maraschini A, et al. The first SARS-CoV-2 wave among pregnant women in Italy: results from a prospective population-based study. *Ann Ist Super Sanita* 2021;57(4):272-85. doi: 10.4415/ANN_21_04_02
3. Donati S, Corsi E, Maraschini A, et al. SARS-CoV-2 infection among hospitalised pregnant women and impact of different viral strains on COVID-19 severity in Italy: a national prospective population-based cohort study. *BJOG* 2022;129(2):221-31. doi: 10.1111/1471-0528.16980 [published Online First: 2021/10/24]
4. Overtoom EM, Rosman AN, Zwart JJ, et al. SARS-CoV-2 infection in pregnancy during the first wave of COVID-19 in the Netherlands: a prospective nationwide population-based cohort study (NethOSS). *BJOG* 2022;129(1):91-100. doi: 10.1111/1471-0528.16903 [published Online First: 20210926]
5. Engjom H, Aabakke AJ, Klungsoyr K, et al. COVID-19 in pregnancy - characteristics and outcomes of pregnant women admitted to hospital because of SARS-CoV-2 infection in the Nordic countries. *Acta Obstet Gynecol Scand* 2021 doi: 10.1111/aogs.14160 [published Online First: 2021/04/23]
6. Varpula R, Ayras O, Aabakke AJM, et al. Early suppression policies protected pregnant women from COVID-19 in 2020: A population-based surveillance from the Nordic countries. *Acta Obstet Gynecol Scand* 2024 doi: 10.1111/aogs.14808 [published Online First: 20240221]
7. Heino AG, M. Nordic Perinatal Statistics 2020. Finland: The Finnish Institute of Health and Welfare (THL), 2022.
