# Supplementary material for: Pregnant women admitted to hospital with covid-19 in 10 European countries: individual patient data meta-analysis of population based cohorts in International Obstetric Survey Systems
Source: BMJ Med. 2024 Dec 12;3(1):e000733. doi: 10.1136/bmjmed-2023-000733 (PMC12164325; doi:10.1136/bmjmed-2023-000733)
Supplement: Supplementary file 4 [file bmjmed-2023-000733.reviewer_comments.pdf]

PEER REVIEW HISTORY

BMJ Medicine publishes all reviews undertaken for accepted manuscripts. Reviewers are asked to complete a checklist review form and are provided with free text boxes to elaborate on their assessment. These free text comments are reproduced below.

ARTICLE DETAILS

**TITLE (PROVISIONAL)**	Lessons for future pandemics: Pregnant women admitted to hospital with covid-19 -an IPD meta-analysis of population-based cohorts in ten European countries within the International Obstetric Survey Systems (INOSS)
**AUTHORS**	Engjom, Hilde; de Bruin, Odette; Ramakrishnan, Rema; Aabakke, Anna; Äyräs, Outi; Deneux-Tharaux, Catherine; Donati, Serena; Knight, Marian; Jonasdottir, Eva; Svanvik, Teresia; Vercoutere, An; Vousden, Nicola; Bloemenkamp, Kitty

VERSION 1 - REVIEW

**REVIEWER NAME**	Pirkle, Catherine McLean
**REVIEWER AFFILIATION**	
**REVIEWER CONFLICT OF INTEREST**	None
**DATE REVIEW RETURNED**	23-Oct-2023

**GENERAL COMMENTS**	This study has three aims: 1) describe the incidence of covid-19 related hospital admissions among pregnant women across 10 countries; 2) describe the incidence of covid-19 severity (at least, this is my understanding from the article); and describe medical treatment provided to pregnant women with moderate to severe covid-19 disease.<br><br>The cross-national analysis, large sample size, and evaluation of covid-19 specific admissions during pregnancy, as well as severity are all novel. The examination of medical treatments provided to pregnant women with moderate to severe disease is particularly novel and the findings very important.<br><br>I have some notable critiques of the article. Discussion of the results and conclusions drawn from them do not align with the results. I will elaborate below.<br><br>Comments:<br>Abstract<br>The methods state that the study design is a descriptive meta-analysis of population-based cohort studies... I am not sure cohort study is accurate. I believe this is just surveillance data form hospital admissions; it is not a longitudinal study.<br><br>The last paragraph of the conclusion focuses on the importance of rapid, robust surveillance systems. The discussion also dedicates a lot of space to discussion surveillance. However, evaluation of surveillance systems or challenges was not an objective of this paper.<br><br>Methods

	Page 8, lines 30-32, under exposure, pregnant women were included if they were admitted to a hospital with a positive SARS-CoV-2 test...up to two days after giving birth. The figure on page 28 only defines inclusion as <=7 days before or during admission. The 2 days after birth is missing.<br><br>Page 9, lines 20-34. These discuss covariates. The term covariate is correct. Later in the article, the term confounder is used instead. Confounder is not correct. The authors do not provide an explanation for their covariate choices. It would be useful to better understand these choices. Also, it is unclear where some of these covariates went when looking at the tables. For example, where is parity in the tables? And gestational age at infection? I would also recommend presenting the variables in the tables in the same order they are described in the text.<br><br>Statistical analyses (page 9, lines 37 on). There are subheadings in this section. These are both helpful and not. I would recommend aligning this section and subheadings according to the articles stated objectives in order to improve clarity.<br><br>Results<br>Page 10, lines 52. There is a word missing between Women and chronic disease.<br><br>No Covid-19 admissions from Iceland. This is surprising. It should be addressed in the discussion. Should Iceland be included in the source population values for calculating incidence?<br><br>Page 14, lines 19-27. This paragraph provides the meta-analysis results for the risk factors. Given the title of the article, these are presumably some of the most important results, because they pool the data from all 10 countries. However, the results are presented in a small paragraph with no corresponding table. It would be much better if these meta-analysis results were presented in a table form, most likely in the supplementary files. The existing Supplementary File 2 provides a good example. It would be useful to see these risks by country, as well the pooled estimate.<br><br>Page 17, Maternal Secondary Outcomes. The authors very briefly describe these results, but they are quite notable. As they indicate, preterm birth before 37 weeks was more common among women with moderate to severe covid-19. The differences between those with moderate-severe covid-19 and mild disease are quite stark (double or more the % for mild disease). The numbers are small and should carefully be interpreted for <34 weeks; even so, the differences again are quite large (e.g., double in France and UK). While I understand that caesarean births included other indications besides covid-19, again there are really important differences between covid-19 severity categories. These should be reported and discussed later.<br><br>The Perinatal Secondary Outcomes do not report on the neonatal ward admissions. There are large enough numbers for this outcomes for interpretation. Those with moderate to severe disease were more likely to have a neonatal ward admission.<br><br>I do not understand why meta-analyses were not run on the secondary outcomes? These are important results and the pooling of data from the 10 countries would have been important for

	understanding risk and helped with the small numbers for certain variables such as birthweight less than 10 percentile or <34 gestational weeks for the maternal outcomes.<br><br>Discussion<br>Relation to other studies: Government responses. The authors describe the Oxford Stringency Index to illustrate implemented measures (I assume covid-19 measures?) and OECD rankings of covid-19 deaths per million inhabitants in 2020. This section is not related to the results at all and I am uncertain which studies they are relating (or how) to their work. While I agree that public health policy differences likely affected the incidence of covid-19 admissions during pregnancy, severity of disease, treatment, and secondary outcomes, these policies were not studied here. If the authors were interested in country policies or other country-level measures on outcomes, there are analyses that allow for such examinations (mixed models and GEE can be used when data are nexted, such as individuals within a country).<br><br>The section on timeliness and data availability is also mis-aligned with the objectives of this paper. The results of this paper are not pertinent to discussing data collection processes. If anything, these considerations should be included in the limitations section.<br><br>I would recommend that the authors organize the Relation to other studies section of the discussion by each of their objectives and in the same order these are presented in the introducation and results.<br><br>This study does not discuss health service and utilization considerations despite a numerator that is based on hospital admissions. The Aday and Andersen papers on health care utilization provide a good starting point for thinking about who may or may not be accessing care and when. Since this paper is examines at length moderate-severe admissions during pregnancy, the discussion should consider/discuss barriers that may have contributed to delayed admissions (both individual and health system). Would any of these have affected incidence estimates or relative risks?<br><br>The authors do not discuss their secondary outcome findings, some of which are important, nor compare them with the existing literature. Given the large sample size and multiple countries in this study, how do the relative risks for these outcomes compare with other studies?<br><br>One of the most important findings of this study is about the use of medications (2nd objective) in pregnant women with covid-19. This is barely discussed. There is a large literature about the need to include pregnant women in trials, for example, as well as their treatment as “vulnerable populations” rather than adults with full agency in their decision-making. Situating these findings in that literature would greatly improve the paper and add policy relevance.

**REVIEWER NAME**	Riley, Richard
**REVIEWER AFFILIATION**	University of Birmingham, Institute of Applied Health Research
**REVIEWER CONFLICT OF INTEREST**	None
**DATE REVIEW RETURNED**	08-Dec-2023

**GENERAL COMMENTS**	This is very interesting meta-analysis summarising the prevalence of outcomes during the pandemic in pregnant women admitted to hospital. It is clear that the authors have worked very hard to undertake this research, and I think it will provide useful information about prognosis in an often neglected patient group, in a pandemic situation, and so will add important value to the medical literature. Some comments from a statistical perspective, to help improve the article before BMJ Medicine can make a decision:<br><br>1) What is not clear is that this is essentially an IPD meta-analysis, where the authors have (unless I am very much mistaken) the raw data for each country. Is this right? If so, it should be labelled as such – as otherwise, readers will think this is a meta-analysis of aggregate (r/n) data obtained from publications. But this is actually an IPD meta-analysis of a selected set of datasets.<br>2) I would suggest to not report I-squared in the abstract. It is a highly misleading measure, and it would be better to focus on tau-squared, which I am pleased to see<br>3) "Amongst 2219 women admitted due to covid-19 in countries with complete respiratory support information, 820 women (39.5%, (95%CI 34.6 to 44.4), I2=47.0%, τ 2=0.0) …" – it does not make sense that tau is zero and I2 is non-zero. There is either an error here, or tau is actually not zero but close to zero (and the studies are all so huge that the value of tau is similar to the within study variation).<br>4) The main conclusion is that 'There was a large variation in incidence …' – to quantify this better (over and above tau squared), a 95% prediction interval would be most useful, to give a range of potential prevalences across settings represented by the 10 studies included in the meta-analysis<br>5) The authors use a two-stage meta-analysis method, but I am concerned about this method for pooling proportions (binomial data). Research shows that it is better to use a one-stage model accounting for clustering, to model the binomial data more directly. This would only give similar results if the data (r/n) are large, as then the two-stage and one-stage methods should give very similar results. Can the authors check that this is indeed the case, and – if so – tell the reader this? Or if not, I would suggest they focus on the one-stage approach. E.g. see https://pubmed.ncbi.nlm.nih.gov/18083461/<br>6) A related comment: 'Due to low numbers of events aggregate data from Denmark, Finland and Norway were combined into a single geographic category.' – this is not necessary in a one-stage model, as it can handle zero or sparse events naturally.<br>7) It's not clear what is actually being pooled in the two-stage approach (logit-p estimates?) and what estimation method (REML?) for the m-a of proportions. For confidence intervals from the meta-analysis, I would expect Hartung-Knapp confidence intervals to allow for uncertainty in the estimation process, but I cannot see this explained. (it is for the sensitivity analysis but not the main analysis, which just say metaprop was used)<br>8) "Heterogeneity across national studies were described using the I2-statistic and tau (τ 2)." – this comes in the section about risk factors, but what about the main analysis of proportions? Also, I2 does not measure heterogeneity, so need to define what it does explain.<br>9) It is not clear why continuous covariates like age are dichotomised when, with the raw data, surely we could model these

	as linear or ideally non-linear associations?<br>10) Following this, it makes no sense to dichotmose age at 35. Why assume people aged 36 and 45 are the same, but people aged 34 and 36 are not? E.g. 'we found an increased risk of moderate to severe disease among women who were ≥35 years when compared to women<35 years (pooled risk ratio 1.32 (95%CI 1.12 to 1.55), with I2=9.4%, p=0.353)' – this makes no sense to me for why this threshold is relevant. Rather model how the whole spectrum of age is associated with changes in the outcome risk.<br>11) The paper loses focus when it starts to adjust for confounders. This turns the paper to more of a causal type study, whereas the heart of the paper is really about summarising outcome risks across different populations<br>12) Supp Table 2 – many proportions are > 1 … something wrong here! E.g. France = 1.64. Perhaps these are not proportions but rather rates per 1000 women, or something like that? Really important to clarify the estimand. Or maybe they are percentages? If so, label as percentages and not proportions.<br><br>I hope these comments are constructive and I look forward to seeing a revision in due course.

**REVIEWER NAME**	Prefumo, Federico
**REVIEWER AFFILIATION**	Istituto Giannina Gaslini Istituto Pediatrico di Ricovero e Cura a Carattere Scientifico
**REVIEWER CONFLICT OF INTEREST**	I participated in data collection for the Italian Obstetric Surveillance System (ItOSS).
**DATE REVIEW RETURNED**	17-Dec-2023

**GENERAL COMMENTS**	This is an interesting meta-analysis of national or regional surveillance data from ten European countries, focusing on covid-19 related hospital admissions in pregnant women.<br><br>The analysis is limited to the pre-vaccine period of the pandemic, so it no longer applies to current practice. However, it identifies themes that are important for the planning of healthcare delivery in case of future public health emergencies.<br><br>The manuscript is well written, the methodology is robust, and the authors appropriately identify and discuss study limitations. A problem with meta-analyzing the different registries is that the information provided and the percentage of missing data were heterogenous due to different protocols/regulations. The authors did their best to overcome this limitation.<br><br>I only have two minor suggestions:<br>1) It is not clear which models were used for meta-analysis of the different outcomes; please specify.<br>2) Please state the fact that universal access to healthcare is provided in the countries/regions from which data originated.

VERSION 1 – AUTHOR RESPONSE

Reviewer: 1
Dr. Catherine McLean Pirkle
Comments to the Author

This study has three aims: 1) describe the incidence of covid-19 related hospital admissions among pregnant women across 10 countries; 2) describe the incidence of covid-19 severity (at least, this is my understanding from the article); and describe medical treatment provided to pregnant women with moderate to severe covid-19 disease.

The cross-national analysis, large sample size, and evaluation of covid-19 specific admissions during pregnancy, as well as severity are all novel. The examination of medical treatments provided to pregnant women with moderate to severe disease is particularly novel and the findings very important.

Authors' response: Thank you for this appreciation.

I have some notable critiques of the article. Discussion of the results and conclusions drawn from them do not align with the results. I will elaborate below.

Comments:
Abstract
The methods state that the study design is a descriptive meta-analysis of population-based cohort studies... I am not sure cohort study is accurate. I believe this is just surveillance data form hospital admissions; it is not a longitudinal study.

Authors' response: We think that the appropriate description of the study design is "a descriptive meta -analysis of population-based cohort studies". For this cohort, pregnant women admitted with a positive SARS-CoV-2 PCR test were prospectively identified from the national or regional source population, and in most countries the source population comprised all women giving birth. Clinical information as well as treatment information and outcome data were retrieved from clinical records, in most countries follow-up information was collected at end of pregnancy for women who were discharged while still pregnant. There were some missing data for women who were discharged with ongoing pregnancy and had their birth at a different obstetric institution in Italy and the Netherlands (as pointed out in the discussion).

The last paragraph of the conclusion focuses on the importance of rapid, robust surveillance systems. The discussion also dedicates a lot of space to discussion surveillance. However, evaluation of surveillance systems or challenges was not an objective of this paper.

Authors response': We agree that learning in order to inform future responses should be more clearly stated in the objective and have revised the description of study aims accordingly. The text on page 6 line 5 now reads: "The aim of the current study was to describe the incidence of covid-19 related admissions in pregnant women, the severity of covid-19 disease, and to describe the medical treatment provided to pregnant women with moderate to severe covid-19 disease, across a range of European countries with different public health containment strategies, with the ultimate aim to inform future responses to similar situations."

Methods
Page 8, lines 30-32, under exposure, pregnant women were included if they were admitted to a

hospital with a positive SARS-CoV-2 test...up to two days after giving birth. The figure on page 28 only defines inclusion as <=7 days before or during admission. The 2 days after birth is missing.

Authors' response: Thank you. the text in Figure 1 has been corrected to include the full definition and now reads: "Inclusion

positive PCR ≤7 days before or during admission up to 2 days after birth»

Page 9, lines 20-34. These discuss covariates. The term covariate is correct. Later in the article, the term confounder is used instead. Confounder is not correct. The authors do not provide an explanation for their covariate choices. It would be useful to better understand these choices.

Authors' response: "Confounder" has been changed to "covariate" in the Methods section as requested. The choice of covariates was informed by existing literature on factors known to be associated with Covid-19 infection and also by the national datasets. This has been clarified in the "Methods" where the section on page 10 from line 9 now reads: "Among pregnant women in the covid -19 admission group in countries where complete information about respiratory support was available (excluding Belgium and Sweden), the relative risk of moderate to severe covid-19 was estimated stratified by selected risk factors identified from the literature and in the national data(15, 18, 32-35);"

Also, it is unclear where some of these covariates went when looking at the tables. For example, where is parity in the tables? And gestational age at infection? I would also recommend presenting the variables in the tables in the same order they are described in the text.

Authors' response: Thank you. Gestational age at infection did not vary much across countries and has been summarized in the text in the first Results paragraph. Parity has also been included in the same paragraph. Neither of these covariates are included in table 1, this is to restrict the number of reported variables to those relevant for the main message as requested by the editor.

Both the text and the subsequent table 1 has been amended to have a similar sequence of covariates. The paragraph on page 11, line 8 now reads: "In the covid-19 admission group the mean (standard deviation) maternal age ranged from 31.0 (4.6) years in Finland to 33.1 (4.6) years in Norway, the median (interquartile range) for gestational age in weeks at admission ranged from 27 (24 to 36) in Sweden and 27 (20 to 32) in Denmark to 34 (28 to 38) in the UK. The proportion of nulliparous women ranged from 20% in Norway and Sweden to 36,5% in the UK and 37,5 % in Finland."

Statistical analyses (page 9, lines 37 on). There are subheadings in this section. These are both helpful and not. I would recommend aligning this section and subheadings according to the articles stated objectives in order to improve clarity.

Authors' response: Thank you for this helpful suggestion, we have removed the subheadings and now describe the analyses in the sequence of the objectives.

In response to reviewer 1 and 2 the section starting on page 9 line 9 now reads:

"*Due to national data protection regulations that restricted international sharing of individual health data a two-stage meta-analysis was used to estimate incidence of covid-19 admissions, the frequencies of moderate to severe infection and medical treatment to pregnant women, by pooling proportions.(25) For this purpose the "metapreg" Stata package for meta-analysis of binomial data with hierarchical modelling was used to estimate the pooled proportion,(26) and with Wilson's estimation of confidence intervals due to small proportions.(27)*

Heterogeneity was assessed using I^2 statistic, τ^2, and prediction intervals. The I^2 statistic used in metapreg is based on the population-parameter based I^2 statistic proposed by Zhou and Dendukuri for binomial data.(28) This statistic takes into account the mean-variance relationship across studies and the sample size of studies in the meta-analysis. Therefore, it tends to perform better than the I^2 statistic proposed by Higgins and Thompson.(29)

Prediction interval as a measure of heterogeneity denotes the range of probability that one can expect in a new study that is similar to the studies included in the meta-analysis.(30)

We stratified incidence by admission group (covid-19 or non-covid). Due to the heterogeneity in the group of non-covid admissions and to avoid misclassification of severe outcomes related to obstetric emergencies in asymptomatic women, the further analyses of covid-19 severity, medical treatment and secondary maternal and perinatal outcomes were reported for women in the covid-19 admission group only."

Results
Page 10, lines 52. There is a word missing between Women and chronic disease.

Authors' response: Thank you. This has been corrected and the text now reads: ". Women with chronic disease prior to pregnancy or gestational disease…

No Covid-19 admissions from Iceland. This is surprising. It should be addressed in the discussion. Should Iceland be included in the source population values for calculating incidence?

Authors' response: Thank you, the low incidence in some countries is a major finding from the study. Pregnant women in Iceland were also part of the source population since active surveillance was implemented at the start of the pandemic. Iceland has a small population and only a few means of entry to the country and thus might have had a better opportunity to control import and internal transmission.

The "race" for large datasets in the international literature has precluded learning from countries with good surveillance and few cases, and this is a main message from the current study. Iceland had a few non-covid admissions, to exclude them from the source population due to zero covid-19 admissions would not be correct.

However, since there were zero covid-19 admissions, Iceland did not contribute any cases to the analyses of severe outcomes, treatment and maternal and perinatal secondary outcomes.

Page 14, lines 19-27. This paragraph provides the meta-analysis results for the risk factors. Given the title of the article, these are presumably some of the most important results, because they pool the data from all 10 countries. However, the results are presented in a small paragraph with no corresponding table. It would be much better if these meta-analysis results were presented in a table form, most likely in the supplementary files. The existing Supplementary File 2 provides a good example. It would be useful to see these risks by country, as well the pooled estimate.

Authors' response: Thank you. Individual maternal covariates/risk factors as identified from the literature and national datasets are described in Table 1, and the tabulated risks by country are shown in the Figure 5 of the submitted manuscript, in addition to the paragraph in the text. Evaluation of individual maternal risk factors was in fact not the most important result, the meta-analysis of the risk factors is particularly relevant to show that differences in risk factors amongst pregnant women in the different countries were not likely to explain the differences in incidence across countries.

Page 17, Maternal Secondary Outcomes. The authors very briefly describe these results, but they are quite notable. As they indicate, preterm birth before 37 weeks was more common among women with moderate to severe covid-19. The differences between those with moderate-severe covid-19 and mild disease are quite stark (double or more the % for mild disease). The numbers are small and should carefully be interpreted for <34 weeks; even so, the differences again are quite large (e.g., double in France and UK). While I understand that caesarean births included other indications besides covid-19, again there are really important differences between covid-19 severity categories. These should be reported and discussed later.

Authors' response:

With a 10-month observation period, we would expect a risk of selection bias towards pregnancies with more severe outcomes and pregnancies ending early. Also, since the Netherlands and Italy had missing information about birth and birth outcomes for women who were discharged still pregnant and later gave birth at a different obstetric institution, the study group originally decided not to run a meta-analysis on the secondary outcomes. We found that a meta-analysis could be misleading, and we have tried to interpret the secondary outcomes with caution. This caution is addressed in the Discussion and have been added in the Results paragraph, while we have included the pooled estimate.

The Results paragraph on page 16 from line 7 now reads: "noting that information about birth was missing for some women discharged with ongoing pregnancies in the Netherlands and Italy. Further details about preterm birth and mode of birth by severity category are shown in supplementary table 4. Amongst women with moderate to severe infection the pooled proportion of preterm birth prior to 34 weeks' gestation was 16.7% ((95%CI 14.3 to 19.4), $\tau^2<0.001$, predictive t interval 12.9 to 21.2), with mild maternal infection the pooled proportion was 3.3% ((95%CI 1.6 to 6.4), $\tau^2=0.2$, predictive t interval 0.9 to 11.1)"

The Perinatal Secondary Outcomes do not report on the neonatal ward admissions. There are large enough numbers for this outcomes for interpretation. Those with moderate to severe disease were more likely to have a neonatal ward admission.

Authors' response:

With the similar caution as discussed above this has been added and the text on page 16 from line 23 now reads: " Amongst infants with available outcome information, neonatal ward admission was more common among infants born to women with moderate to severe infection; the pooled proportion of neonatal ward admission with moderate to severe infection was 32.8% (95%CI 25.9 to 40.5, τ^2=0.09, predictive t interval 20.9 to 47.4) versus mild infection 13.3% (95%CI 11.7 to 15.3, τ^2<0.0001, predictive t interval 10.6 to 16.5)"

I do not understand why meta-analyses were not run on the secondary outcomes? These are important results and the pooling of data from the 10 countries would have been important for understanding risk and helped with the small numbers for certain variables such as birthweight less than 10 percentile or <34 gestational weeks for the maternal outcomes.

Authors' response: With a 10-month observation period, we would expect a risk of selection bias towards pregnancies with more severe outcomes and pregnancies ending early. Also, since the Netherlands and Italy had missing information about birth and birth outcomes for women who were discharged still pregnant and later gave birth at a different obstetric institution, the study group originally decided not to run a meta-analysis on the secondary outcomes. We found that a meta-analysis could be misleading, and we have tried to interpret the secondary outcomes with caution. This caution is addressed in the Discussion and have been added in the Results paragraph, while we have included the pooled estimate as suggested from the reviewer.

Further, we reasoned that birthweight was not likely to be affected by SARS-CoV-2 infection for women with a current infection who ended their pregnancy at the time of admission (up to seven days of exposure to the virus). This could be an important factor to address in future studies of outcomes for women who continued their pregnancies, but was beyond the scope of the current analyses.

In response to the reviewer comments and suggestion, we have added pooled frequencies of preterm birth prior to 34 weeks' gestation by severity of infection to the paragraph reporting secondary maternal outcomes and pooled neonatal ward admission frequency by severity of maternal infection to the paragraph reporting secondary perinatal outcomes.

Discussion

Relation to other studies: Government responses. The authors describe the Oxford Stringency Index to illustrate implemented measures (I assume covid-19 measures?) and OECD rankings of covid-19 deaths per million inhabitants in 2020. This section is not related to the results at all and I am uncertain which studies they are relating (or how) to their work.

Authors' response: This section was meant to show that the differences in incidence observed for pregnant women is also mirrored in population surveillance of hospital admissions and deaths in the general population. The sequence of the Discussion paragraphs and the text has been modified to clarify this.

The paragraph on page 17, starting from line 8 now reads:

“While few studies have reported population-based estimates for incidence of covid-19 admission in the pregnant population, other population-based sources do describe a similar distribution of severe covid-19 cases and covid-deaths across countries. Using the Organisation for Economic Co-operation and Development(OECD) and European Centre for Disease Control (ECDC) as sources, the Nordic countries Denmark, Finland and Norway were among the countries with fewest deaths per million inhabitants in 2020, and Sweden, France, United Kingdom, Italy and Belgium among the countries with highest mortality.(8) The latter countries also had the highest estimates of total population infected.(45) When using the Oxford stringency index as a summary description of government covid-19 responses, a high early index was associated with reduced mortality.(44) According to the government response tracker used for the SI, Italy was the first to implement measures resulting in an index >50 on 24 February, and the United Kingdom and Sweden last, on 21 March and 26 March, respectively.(44) The other four Nordic countries reached a SI of >50 on 13 to 17 March.”

While I agree that public health policy differences likely affected the incidence of covid-19 admissions during pregnancy, severity of disease, treatment, and secondary outcomes, these policies were not studied here. If the authors were interested in country policies or other country-level measures on outcomes, there are analyses that allow for such examinations (mixed models and GEE can be used when data are nexted, such as individuals within a country).

Authors’ response: We thank the reviewer for this very good point. However, there is evidence to suggest that the policies played a role, since the differences in incidence cannot be explained by differences in risk factors for covid in the pregnant populations. We strongly agree that this should be pursued in future studies using different analytical approaches and looking into the impact of specific policies.

The section on timeliness and data availability is also mis-aligned with the objectives of this paper. The results of this paper are not pertinent to discussing data collection processes. If anything, these considerations should be included in the limitations section.

Authors’ response: In response to the reviewer’s very good suggestion we have rearranged the sequence of the discussion of study results in relation to other studies. We have moved the lessons learned about surveillance to implications for policy-makers and clinicians.

I would recommend that the authors organize the Relation to other studies section of the discussion by each of their objectives and in the same order these are presented in the introduction and results.

Authors’ response: Thank you for a very good suggestion, we have changed the sequence in the text accordingly.

This study does not discuss health service and utilization considerations despite a numerator that is based on hospital admissions. The Aday and Andersen papers on health care utilization provide a good starting point for thinking about who may or may not be accessing care and when. Since this paper is examines at length moderate-severe admissions during pregnancy, the discussion should consider/discuss barriers that may have contributed to delayed admissions (both individual and health system). Would any of these have affected incidence estimates or relative risks?

Authors' response: We have discussed this topic briefly, particularly in relation to generalizability (page 20 from line 18) and also in the paragraph discussing testing strategies (page 16, line 9-14) and in relation to the universal access to health care during pregnancy in the participating countries (page 7, line 19).

The authors do not discuss their secondary outcome findings, some of which are important, nor compare them with the existing literature. Given the large sample size and multiple countries in this study, how do the relative risks for these outcomes compare with other studies?

Authors' response: Given the caveats discussed in responses to the prior comments about the secondary outcomes, the aim of the current study needs to reflect the main study aims. We have added a brief paragraph about the secondary outcomes in the discussion.

*The text on page 19 from line 27 now reads: "*Maternal and perinatal outcomes were secondary outcomes in the current study, the observation time was relatively short and information about birth and infant outcomes were lacking in some countries for women who were discharged with an on-going pregnancy and gave birth in a different hospital. However, the available information about outcomes from the index admission could point to a higher risk of preterm birth, caesarean birth and neonatal ward admission among women with moderate to severe infection. While the current study cannot offer conclusive evidence, these results indicate the merit of nuanced analyses taking severity of covid-19 into account when assessing maternal and perinatal outcomes in future studies."

One of the most important findings of this study is about the use of medications (2nd objective) in pregnant women with covid-19. This is barely discussed. There is a large literature about the need to include pregnant women in trials, for example, as well as their treatment as "vulnerable populations" rather than adults with full agency in their decision-making. Situating these findings in that literature would greatly improve the paper and add policy relevance.

Authors' response: Thank you, the text has been amended to include this and the paragraph in the discussion on page 19, from line 21 now reads: "During the first and second wave of the pandemic pregnant women with moderate to severe covid-19 did not receive covid-19 directed medical treatment, even when there were few safety issues and after the RECOVERY trial indicated a reduction of severe morbidity and death with early corticosteroid treatment in adults needing respiratory support.(55) However, the apparent undertreatment aligns with previous experiences with epidemic threats with persisting bias regarding vaccine development and vaccination,(16) medical treatment(56) and also in development of clinical guidelines."

Reviewer: 2
Prof. Richard Riley, University of Birmingham
Comments to the Author
This is very interesting meta-analysis summarising the prevalence of outcomes during the pandemic in pregnant women admitted to hospital. It is clear that the authors have worked very hard to undertake this research, and I think it will provide useful information about prognosis in an often neglected patient group, in a pandemic situation, and so will add important value to the medical

literature. Some comments from a statistical perspective, to help improve the article before BMJ Medicine can make a decision:

Authors' response: Thank you for the appreciation.

1) What is not clear is that this is essentially an IPD meta-analysis, where the authors have (unless I am very much mistaken) the raw data for each country. Is this right? If so, it should be labelled as such – as otherwise, readers will think this is a meta-analysis of aggregate (r/n) data obtained from publications. But this is actually an IPD meta-analysis of a selected set of datasets.

Authors' response: Individual data were indeed available in each country, and we conducted a two-step meta-analysis due to GDPR regulations. We have revised the text to clarify this in the title, abstract, and the in the first paragraph of the statistical analysis section. The text on page 9, line 9 onwards now reads: "Due to national data protection restrictions for sharing of individual health data, a two-stage individual participant data (IPD) meta-analysis was used to estimate incidence of covid-19 admissions, the frequencies of moderate to severe infection and medical treatment to pregnant women, by pooling proportions.(25)"

2) I would suggest to not report I-squared in the abstract. It is a highly misleading measure, and it would be better to focus on tau-squared, which I am pleased to see.

Authors' response: Thanks, we have amended the abstract text accordingly.

3) "Amongst 2219 women admitted due to covid-19 in countries with complete respiratory support information, 820 women (39.5%, (95%CI 34.6 to 44.4), I2=47.0%, τ 2=0.0) …" – it does not make sense that tau is zero and I2 is non-zero. There is either an error here, or tau is actually not zero but close to zero (and the studies are all so huge that the value of tau is similar to the within study variation).

Authors' response: We redid the analyses to obtain more decimal points and the tau-squared was 0.02, the text on page 13, line 16-19 has been amended and now reads: " Amongst women in the covid-19 admission group 38.9% had moderate to severe infection (95%CI 34.6 to 43.3), I^2=46.6%, τ^2=0.02, 95% predictive t interval 31.9 to 46.3)"

4) The main conclusion is that 'There was a large variation in incidence …' – to quantify this better (over and above tau squared), a 95% prediction interval would be most useful, to give a range of potential prevalences across settings represented by the 10 studies included in the meta-analysis

Authors' response: Thank you, we have added a description of prediction intervals in the methods section, the estimated prediction intervals have been added to text, the forest plots in figures, and in supplementary table 2.

5) The authors use a two-stage meta-analysis method, but I am concerned about this method for pooling proportions (binomial data). Research shows that it is better to use a one-stage model accounting for clustering, to model the binomial data more directly. This would only give similar results if the data (r/n) are large, as then the two-stage and one-stage methods should give very similar results. Can the authors check that this is indeed the case, and – if so – tell the reader this? Or if not, I would suggest they focus on the one-stage approach. E.g. see https://pubmed.ncbi.nlm.nih.gov/18083461/

Authors' response: We agree, but with the European Data Protection Regulation (GDPR) and strict national interpretations of this, many countries could not share individual health information and consequently analyses had to be done in a federated approach with a two-stage design.

6) A related comment: 'Due to low numbers of events aggregate data from Denmark, Finland and Norway were combined into a single geographic category.' – this is not necessary in a one-stage model, as it can handle zero or sparse events naturally.

Authors' response: We agree, but again, sharing of individual data about a few individuals was not allowed. We even had to combine the number of events to across countries comply with regulations for potential identifiability. The challenge was not the number of events in the statistical model, but the national regulations for reporting of rare events.

We have amended the text to clarify this on page 8, lines 9 to 15:

"Due to national data protection restrictions for sharing of individual health data a two-stage individual participant data (IPD) meta-analysis was used to estimate incidence of covid-19 admissions, the frequencies of moderate to severe infection and medical treatment to pregnant women, by pooling proportions.(25)"

and page 9, lines 14-16: "Due to low numbers of events and concerns for identifiability according to national data protection regulations for health data, aggregate data from Denmark, Finland and Norway were combined into a single geographic category."

7) It's not clear what is actually being pooled in the two-stage approach (logit-p estimates?)

Authors' response:

For the meta-analysis we obtained counts (not logit-p) from each country and pooled proportions and pooled risk ratios were estimated. Metapreg does the meta-analysis in one step using aggregate data, not a two-step meta-analysis is where the standard errors in each study are computed, and, in the second step, a weighted regression model is fitted with the weights computed from the standard

errors computed earlier. Metapreg is based on logistic regression where the first step is not necessary.

The text has been amended to clarify this on page 8 line 9-15 as referred to in the response above.

-and what estimation method (REML?) for the m-a of proportions.

Authors' response: the STATA metapreg command uses logistic regression (maximum likehood optimization) with marginal standization for the pooled proportion estimates.

According to the authors of the metapreg syntax (Nyaga and Arbyn), this method uses hierarchical modeling approach with an iterative process to estimate the pooled proportion.

For confidence intervals from the meta-analysis, I would expect Hartung-Knapp confidence intervals to allow for uncertainty in the estimation process, but I cannot see this explained. (it is for the sensitivity analysis but not the main analysis, which just say metaprop was used)

Authors' response: "Metapreg" was used for the main analyses, but "metaprop" results with Freeman-Tuckey double arcsine transformation were reported in the supplementary since this has been the standard method for pooling proportions. We used Wilsons method for both meta-analyses of proportions to estimate confidence intervals to aid comparability with the "metapreg" analyses. Hartung-Knapp-Sidik-Jonkman variance estimator was used to estimate confidence intervals for the pooled risk ratio estimates.

This has been clarified in the manuscript in the methods section which now reads from page 9, line 12: ",(25) *For this purpose the "metapreg" Stata package for meta-analysis of binomial data with hierarchical modelling was used to estimate the pooled proportions,(26) and with Wilson's estimation of confidence intervals due to small proportions.(27)".*

And on page 10, from line 6-13:

"Among pregnant women in the covid-19 admission group in countries where complete information about respiratory support was available (excluding Belgium and Sweden), the risk ratio for moderate to severe covid-19 was estimated stratified by selected risk factors identified from the literature and in the national data(15, 18, 32-35);; (maternal age (≥35 years vs < 35 years), obesity (obese vs not obese), and migrant or minority background (maternal country of birth outside Europe vs European born, or minority (BAME) background vs White)) using a random effect maximum likelihood (REML) model with Hartung-Knapp-Sidik-Jonkman (HKSJ) variance estimator.(36, 37)"

8) “Heterogeneity across national studies were described using the I2-statistic and tau (τ 2).” – this comes in the section about risk factors, but what about the main analysis of proportions? Also, I2 does not measure heterogeneity, so need to define what it does explain.

Authors’ response:

In response to comments from all reviewers the text has been amended to clarify this for all main outcome analyses; incidence, disease severity including risk factors and proportion of women receiving medical treatment. Further, prediction intervals for the pooled proportions have been added. The I2 statistic used in “metapreg” is by Zhou et al which takes better into account the sample size of the national studies by accounting for the mean-variance relationship across studies (Zhou and Dendukuri, 2014).

This section in methods, page 9 from line 16 now reads: “Heterogeneity was assessed using I^2 statistic, τ^2, and prediction intervals. The I^2 statistic used in metapreg is based on a population-parameter based I^2 statistic proposed by Zhou and Dendukuri for binomial data.(28) This statistic takes into account the mean-variance relationship across studies and the sample size of studies in the meta-analysis. Therefore, it tends to perform better than the I^2 statistic proposed by Higgins and Thompson.(29) Prediction interval as a measure of heterogeneity denotes the range of probability that one can expect in a new study that is similar to the studies included in the meta-analysis.(30)”

9) It is not clear why continuous covariates like age are dichotomised when, with the raw data, surely we could model these as linear or ideally non-linear associations?

Authors’ response: In the adjusted risk ratio models age was treated as a continuous covariate.

10) Following this, it makes no sense to dichtomise age at 35. Why assume people aged 36 and 45 are the same, but people aged 34 and 36 are not? E.g. ‘we found an increased risk of moderate to severe disease among women who were ≥35 years when compared to women<35 years (pooled risk ratio 1.32 (95%CI 1.12 to 1.55), with I2=9.4%, p=0.353)’ – this makes no sense to me for why this threshold is relevant. Rather model how the whole spectrum of age is associated with changes in the outcome risk.

Authors’ response: The threshold has been identified based on the literature and the previous publications from the participating countries, with UK representing the largest dataset. Further, the GDPR regulations in countries with small datasets required few categories. We have clarified this in the methods, also in response to reviewer 1, this section on page 10 from line 6 now reads: “Among pregnant women in the covid-19 admission group in countries where complete information about respiratory support was available (excluding Belgium and Sweden), the risk ratio for moderate to severe covid-19 was estimated stratified by selected risk factors identified from the literature and in the national data (15, 18, 32-35);”

11) The paper loses focus when it starts to adjust for confounders. This turns the paper to more of a causal type study, whereas the heart of the paper is really about summarising outcome risks across different populations

Authors' response: Thank you. The text has been modified to clarify this, also in response to the comments and specific suggestions from Reviewer 1 addressed above. We hope the new organization of results and discussion communicates a clear message.

12) Supp Table 2 – many proportions are > 1 … something wrong here! E.g. France = 1.64. Perhaps these are not proportions but rather rates per 1000 women, or something like that? Really important to clarify the estimand. Or maybe they are percentages? If so, label as percentages and not proportions.

Authors' response: Thank you, the proportions are incidence per 1000 maternities, the labels have been corrected

I hope these comments are constructive and I look forward to seeing a revision in due course.
Best wishes, Prof Richard Riley
Chief Statistics Editor

Reviewer: 3
Prof. Federico Prefumo, Istituto Giannina Gaslini Istituto Pediatrico di Ricovero e Cura a Carattere Scientifico

Comments to the Author
This is an interesting meta-analysis of national or regional surveillance data from ten European countries, focusing on covid-19 related hospital admissions in pregnant women.

The analysis is limited to the pre-vaccine period of the pandemic, so it no longer applies to current practice. However, it identifies themes that are important for the planning of healthcare delivery in case of future public health emergencies.

The manuscript is well written, the methodology is robust, and the authors appropriately identify and discuss study limitations. A problem with meta-analyzing the different registries is that the information provided and the percentage of missing data were heterogenous due to different protocols/regulations. The authors did their best to overcome this limitation.

Authors' response: Thank you, we appreciate the feedback.

I only have two minor suggestions:
1) It is not clear which models were used for meta-analysis of the different outcomes; please specify.

Authors' response: Also in response to specific comments from all reviewers, we have amended the text to clarify this as outlined above.

2) Please state the fact that universal access to healthcare is provided in the countries/regions from which data originated.

Authors' response:

Short information about the obstetric health care in participating countries have been added in the description of the study population on page 7 line 19-20, as context information regarding test policies, and in discussion about threshold for admission to hospital on page 16, lines 9 -13

VERSION 2 – REVIEW

**REVIEWER NAME**	Riley, Richard
**REVIEWER AFFILIATION**	University of Birmingham, Institute of Applied Health Research
**REVIEWER CONFLICT OF INTEREST**	None
**DATE REVIEW RETURNED**	03-Mar-2024

**GENERAL COMMENTS**	I thank the authors for their revision and response.<br><br>I do have some remaining comments.<br><br>Firstly, there is still confusion about the meta-analysis method used. That is, the authors say they used a two-stage approach, but I think they do use a one-stage approach as I wanted. They are not consistent in their response, e.g.<br><br>'Due to national data protection restrictions for sharing of individual health data a two-stage<br>individual participant data (IPD) meta-analysis was used to estimate incidence of covid-19<br>admissions, the frequencies of moderate to severe infection and medical treatment to<br>pregnant women, by pooling proportions'<br><br>- so this says a two-stage approach was used. But then:<br><br>'For the meta-analysis we obtained counts (not logit-p) from each country and pooled<br>proportions and pooled risk ratios were estimated. Metapreg does the meta-analysis in one<br>step using aggregate data, not a two-step meta-analysis is where the standard errors in each<br>study are computed, and, in the second step, a weighted regression model is fitted with the<br>weights computed from the standard errors computed earlier. Metapreg is based on logistic<br>regression where the first step is not necessary'<br><br>- so this suggests a one-step approach was used.<br><br>This confusion is that in their 'first' step the authors are calculating r

	and n for each country. These are then pooled in a one-stage model (that is, it is NOT the proportions themselves being pooled but the r and n in a one-stage logistic regression model allowing for clustering). So yes, there are two stages to do the meta-analysis (1st stage derive r and n for each study, then 2nd stage use a one-stage model). but the actual model is a one-stage model that synthesises r/n (and so does not derive estimates and standard errors first before then pooling). This is important, because the one-stage models being fitted are more appropriate, as they model the binomial nature of the data directly, for example without needing continuity corrections to deal with zero events and so forth. Please can the authors therefore clarify they used one-stage models, not two-stage models to do the actual analysis, using r and n values for each country. Though note, I think the risk ratio models did use a two-stage modelling approach.<br><br>When introducing I-squared in the methods, there is now a detailed description, but without a definition. Please add' I-squared measures the percentage of total variance in the study estimates that is due to between-study heterogeneity'<br><br>Thank you for clarifying that the results are presented as X per 1000 maternities. This is not a proportion (bounded between 0 and 1), which contradicts the methods sections about pooling proportions. So again, how was the meta-analysis done (or transformed afterwards) to put it on the per 1000 scale?<br><br>I still don't agree with the categorisation of age. The authors say the threshold for age was 'identified based on the literature and the previous publications' – this is still not any strong statistical justification for losing information. I suggest that they add a limitation to the discussion section to state that the threshold is arbitrary and age would be better modelled as a continuous covariate in the future.<br><br>The conclusions say that there is a 'striking variation' is risk – yet the results suggest 'ranging from no admissions in Iceland to 1.7 in France and 1.9 the UK' – is there really a 'striking' difference from 0 to 1.9 per 1000?<br><br>The paper says 'As shown in table 1, the distribution of maternal characteristics varied across countries. In meta-analyses stratified by age, obesity, and migrant/minority background we found an increased risk of moderate to severe disease among women who were ≥35 years when compared to women<35 years (pooled risk ratio 1.32 (95%CI 1.12 to 1.55), with I2=9.4%, p=0.353), for obese compared to non-obese women (pooled risk ratio 1.60 (1.44 to 1.78), with I2=0%, p=0.744)'<br><br>- the CIs are all excluding the null value of 1, and yet the p-values are much > 0.05. Something is amiss here.<br><br>In summary, in the context of an improved revision, there are still a few areas to iron out before BMJ Medicine can proceed with this interesting paper.

VERSION 2 – AUTHOR RESPONSE

Reviewer: 2
Prof. Richard Riley, University of Birmingham
Comments to the Author
I thank the authors for their revision and response.

I do have some remaining comments.

Firstly, there is still confusion about the meta-analysis method used. That is, the authors say they used a two-stage approach, but I think they do use a one-stage approach as I wanted. They are not consistent in their response, e.g.

'Due to national data protection restrictions for sharing of individual health data a two-stage individual participant data (IPD) meta-analysis was used to estimate incidence of covid-19 admissions, the frequencies of moderate to severe infection and medical treatment to pregnant women, by pooling proportions'

- so this says a two-stage approach was used. But then:

'For the meta-analysis we obtained counts (not logit-p) from each country and pooled proportions and pooled risk ratios were estimated. Metapreg does the meta-analysis in one step using aggregate data, not a two-step meta-analysis is where the standard errors in each study are computed, and, in the second step, a weighted regression model is fitted with the weights computed from the standard errors computed earlier. Metapreg is based on logistic regression where the first step is not necessary'

- so this suggests a one-step approach was used.

This confusion is that in their 'first' step the authors are calculating r and n for each country. These are then pooled in a one-stage model (that is, it is NOT the proportions themselves being pooled but the r and n in a one-stage logistic regression model allowing for clustering). So yes, there are two stages to do the meta-analysis (1st stage derive r and n for each study, then 2nd stage use a one-stage model). but the actual model is a one-stage model that synthesises r/n (and so does not derive estimates and standard errors first before then pooling). This is important, because the one-stage models being fitted are more appropriate, as they model the binomial nature of the data directly, for example without needing continuity corrections to deal with zero events and so forth. Please can the authors therefore clarify they used one-stage models, not two-stage models to do the actual analysis, using r and n values for each country. Though note, I think the risk ratio models did use a two-stage modelling approach.

Response: Thank you for these questions for clarification, we agree that the description was not concise and clear. We have revised the description of the analyses to clarify that one-stage meta-analysis was used to for estimation of pooled incidence of covid admission, severity of disease, of medical treatment, and secondary maternal and perinatal outcomes. We have also added that the two -stage model was used to estimate pooled risk ratio analyses.

When introducing I-squared in the methods, there is now a detailed description, but without a definition. Please add' I-squared measures the percentage of total variance in the study estimates that is due to between-study heterogeneity'

Response: We have revised the methods paragraph describing heterogeneity to include this statement.

Thank you for clarifying that the results are presented as X per 1000 maternities. This is not a

proportion (bounded between 0 and 1), which contradicts the methods sections about pooling proportions. So again, how was the meta-analysis done (or transformed afterwards) to put it on the per 1000 scale?

Response: Thank you for this clarification. In the meta-analyses of incidence we directly used the power option in STATA to get the results on a per 1000 scale. This has been added in the paragraph.

I still don't agree with the categorisation of age. The authors say the threshold for age was 'identified based on the literature and the previous publications' – this is still not any strong statistical justification for losing information. I suggest that they add a limitation to the discussion section to state that the threshold is arbitrary and age would be better modelled as a continuous covariate in the future.

Response: We agree, have noted this as a point of learning for future analyses and added this in the discussion of limitations. The paragraph now reads:

"Further, regarding the available covariates, age was categorised in the current study based on published research and national results. The cut-off at 35 years is however arbitrary and age would be better modelled as a continuous covariate for future studies. While migrant background is not equivalent to minority background we had to adopt to different practices in recording and reporting this across countries"

The conclusions say that there is a 'striking variation' is risk – yet the results suggest 'ranging from no admissions in Iceland to 1.7 in France and 1.9 the UK' – is there really a 'striking' difference from 0 to 1.9 per 1000?

Response: From a clinical perspective the difference of incidence of covid admission, with an almost 10-fold higher risk in the UK compared to Norway and Finland, is striking. This was also reflected in the impact of covid-19 on maternal mortality in the UK, as covid-19 became the leading single cause of maternal death during the triennium from 2019 to 2021. However, in keeping with good practice for scientific language and reporting we have removed the adjective from the text and leave the interpretation to the reader.

The paper says 'As shown in table 1, the distribution of maternal characteristics varied across countries. In meta-analyses stratified by age, obesity, and migrant/minority background we found an increased risk of moderate to severe disease among women who were ≥35 years when compared to women<35 years (pooled risk ratio 1.32 (95%CI 1.12 to 1.55), with I2=9.4%, p=0.353), for obese compared to non-obese women (pooled risk ratio 1.60 (1.44 to 1.78), with I2=0%, p=0.744)'

- the CIs are all excluding the null value of 1, and yet the p-values are much > 0.05. Something is amiss here.

Response: We recognise that the included p-value, which is related to the heterogeneity measures and not to the risk ratio estimates, is confusing, and it does not add anything to the description of heterogeneity. We have removed the p-values. This section now reads:

"In meta-analyses stratified by age, obesity, and migrant/minority background we found an increased risk of moderate to severe disease among women who were ≥35 years when compared to women<35 years (pooled risk ratio 1.32 (95%CI 1.12 to 1.55), with I^2=9.4%), for obese compared to non-obese women (pooled risk ratio 1.60 (1.44 to 1.78), with I^2=0%), and for women with migrant or minority background compared to women with White (majority) or European-born background (pooled risk ratio of 1.21 (0.97 to 1.51), with I^2= 40.8) (figure 5)."

In summary, in the context of an improved revision, there are still a few areas to iron out before BMJ

Medicine can proceed with this interesting paper. Best wishes, Prof Richard Riley

Response: Thank you for the patience and good advice. We have tried to remove the "last creases" and see that this has improved the clarity of the manuscript.

VERSION 3 – REVIEW

**REVIEWER NAME**	Riley, Richard
**REVIEWER AFFILIATION**	University of Birmingham, Institute of Applied Health Research
**REVIEWER CONFLICT OF INTEREST**	None
**DATE REVIEW RETURNED**	05-Jun-2024

**GENERAL COMMENTS**	The authors response is very clear and the revisions appropriate. My only remaining suggestions are:<br><br>1) to include the estimated tau-squared too, alongside the I2 estimate, in the brackets of the results section where omitted.<br><br>e.g. 'In meta-analyses stratified by age, obesity, and migrant/minority background we found an increased risk of moderate to severe disease among women who were ≥35 years when compared to women<35 years (pooled risk ratio 1.32 (95%CI 1.12 to 1.55), with I2=9.4% and tau2 of XXX), …'<br><br>2) The abstract results includes this statement: 'At most, one in six women with moderate to severe covid-19 received SARS-CoV-2 directed medical treatment, although two of three were given thromboprophylaxis.' – but why not provide the pooled result and CI and tau-squared for this result? It seems the 1 in 6 might be an unweighted result by combining all the data assuming one big study?<br><br>3) The abstract conclusion is that 'There is a strong indication that national public health policies played a substantial and previously unrecognised role in protecting pregnant women.' – but this is not backed-up by the results in the abstract. The results show heterogeneity, but that could be due to different covid19 prevalences in each country due to natural variation due to onset of new variants, not the national health policies per se?<br><br>4) 'In meta-analyses stratified by age, obesity, and migrant/minority background we found an increased risk of moderate to severe disease among women who were ≥35 years when compared to women<35 years (pooled risk ratio 1.32 (95%CI 1.12 to 1.55), with I2=9.4%), for obese compared to non-obese women (pooled risk ratio 1.60 (95% CI 1.44 to 1.78), with I2=0%), and for women with migrant or minority background compared to women with White (majority) or European-born background (pooled risk ratio of 1.21 (95% CI 0.97 to 1.51), with I2= 40.8) (figure 5)." – this should make is clearer that the stratification is at the study level, and not the patient level, which is a critical difference as there is the potential for study-level confounding. E.g. change to say:<br><br>"In meta-analyses OF STUDIES stratified by age, obesity, and

	migrant/minority background we found an increased risk of moderate to severe disease IN STUDIES OF women who were ≥35 years when compared to STUDIES OF women<35 years (pooled risk ratio 1.32 (95%CI 1.12 to 1.55), with I2=9.4%), for STUDIES OF obese compared to STUDIES OF non-obese women (pooled risk ratio 1.60 (95% CI 1.44 to 1.78), with I2=0%), and for STUDIES OF women with migrant or minority background compared to STUDIES OF women with White (majority) or European-born background (pooled risk ratio of 1.21 (95% CI 0.97 to 1.51), with I2= 40.8) (figure 5).” – hope that makes sense and I am not missing something.<br><br>I look forward to this being published in the BMJ Medicine

VERSION 3 – AUTHOR RESPONSE

Reviewer: 2
Prof. Richard Riley, University of Birmingham
Comments to the Author
The authors response is very clear and the revisions appropriate.
My only remaining suggestions are:

1) to include the estimated tau-squared too, alongside the I2 estimate, in the brackets of the results section where omitted.

e.g. ‘In meta-analyses stratified by age, obesity, and migrant/minority background we found an increased risk of moderate to severe disease among women who were ≥35 years when compared to women<35 years (pooled risk ratio 1.32 (95%CI 1.12 to 1.55), with I2=9.4% and tau2 of XXX), …’

Authors’ response: We have added this information in the revised manuscript. As illustrated by the distribution in the forest plot the tau-squared values were very low. The full text of this paragraph is now outlined in response to the reviewers point 4.

2) The abstract results includes this statement: ‘At most, one in six women with moderate to severe covid-19 received SARS-CoV-2 directed medical treatment, although two of three were given thromboprophylaxis.’ – but why not provide the pooled result and CI and tau-squared for this result? It seems the 1 in 6 might be an unweighted result by combining all the data assuming one big study?
Authors’ response: We have added the pooled proportions as suggested.
The abstract text now reads: “At most, 16.8% (95%CI 7.7 to 32.9, I2=81.8, τ2=0.7) of women with moderate to severe covid-19 received SARS-CoV-2 directed medical treatment with corticosteroids, although 66.6% (95%CI 59.4 to 73.2) I2=50.1, τ2=0.06) were given thromboprophylaxis.”

3) The abstract conclusion is that ‘There is a strong indication that national public health policies played a substantial and previously unrecognised role in protecting pregnant women.’ – but this is not backed-up by the results in the abstract. The results show heterogeneity, but that could be due to different covid19 prevalences in each country due to natural variation due to onset of new variants, not the national health policies per se?
Authors’ response: The dominant variant throughout the study period of 2020 was the wildtype variant in all the countries. While the abstract does not allow for repeating the whole discussion section from the manuscript, the conclusion is drawn after taking other evidence into consideration. The difference in incidence in the results section of the abstract back up this conclusion.
We have amended the abstract text to clarify: “Population-based surveillance in ten European countries from the first ten months of the pandemic showed variation in pregnant womens’ risk of covid-19 admission. This indicates that national public health policies played a substantial and

previously unrecognised role in protecting pregnant women."

4) 'In meta-analyses stratified by age, obesity, and migrant/minority background we found an increased risk of moderate to severe disease among women who were ≥35 years when compared to women<35 years (pooled risk ratio 1.32 (95%CI 1.12 to 1.55), with I2=9.4%), for obese compared to non-obese women (pooled risk ratio 1.60 (95% CI 1.44 to 1.78), with I2=0%), and for women with migrant or minority background compared to women with White (majority) or European-born background (pooled risk ratio of 1.21 (95% CI 0.97 to 1.51), with I2= 40.8) (figure 5)." – this should make is clearer that the stratification is at the study level, and not the patient level, which is a critical difference as there is the potential for study-level confounding. E.g. change to say:

"In meta-analyses OF STUDIES stratified by age, obesity, and migrant/minority background we found an increased risk of moderate to severe disease IN STUDIES OF women who were ≥35 years when compared to STUDIES OF women<35 years (pooled risk ratio 1.32 (95%CI 1.12 to 1.55), with I2=9.4%), for STUDIES OF obese compared to STUDIES OF non-obese women (pooled risk ratio 1.60 (95% CI 1.44 to 1.78), with I2=0%), and for STUDIES OF women with migrant or minority background compared to STUDIES OF women with White (majority) or European-born background (pooled risk ratio of 1.21 (95% CI 0.97 to 1.51), with I2= 40.8) (figure 5)." – hope that makes sense and I am not missing something.

Authors' response: We agree that it is important to be clear about level of analysis and stratification, but at the same time it is relevant for the reader to know that data about the women with/without these risk factors were collected in the same national cohorts, not from separate studies. We have rephrased this paragraph to highlight that these results are from country level analyses:

"In meta-analyses stratified by age, obesity, and migrant/minority background at country level we found an increased risk of moderate to severe disease among women who were ≥35 years when compared to women<35 years (pooled risk ratio 1.32 (95%CI 1.12 to 1.55), with I2=9.4%, τ2 <0.001), for obese compared to non-obese women (pooled risk ratio 1.60 (95% CI 1.44 to 1.78), with I2=0%, τ2 <0.001), and for women with migrant or minority background compared to women with White (majority) or European-born background (pooled risk ratio of 1.21 (95% CI 0.97 to 1.51), with I2= 40.8, τ2 =0.003) (figure 5)."

I look forward to this being published in the BMJ Medicine
